# Clinical specificity profile for novel rapid acting antidepressant drugs

**DOI:** 10.1097/YIC.0000000000000488

**Published:** 2023-06-30

**Authors:** Mauro Scala, Giuseppe Fanelli, Diana De Ronchi, Alessandro Serretti, Chiara Fabbri

**Affiliations:** a Department of Biomedical and Neuromotor Sciences, University of Bologna, Bologna, Italy; b Department of Human Genetics, Radboud University Medical Center, Donders Institute for Brain, Cognition and Behaviour, Nijmegen, The Netherlands; c Social, Genetic & Developmental Psychiatry Centre, Institute of Psychiatry, Psychology & Neuroscience, King’s College London, London, UK

**Keywords:** antidepressants, bipolar depression, clinical trials, gamma-aminobutyric acid, major depressive disorder, novel rapid acting antidepressant, pharmacodynamic mechanisms, postpartum depression, treatment-resistant depression

## Abstract

Mood disorders are recurrent/chronic diseases with variable clinical remission rates. Available antidepressants are not effective in all patients and often show a relevant response latency, with a range of adverse events, including weight gain and sexual dysfunction. Novel rapid agents were developed with the aim of overcoming at least in part these issues. Novel drugs target glutamate, gamma-aminobutyric acid, orexin, and other receptors, providing a broader range of pharmacodynamic mechanisms, that is, expected to increase the possibility of personalizing treatments on the individual clinical profile. These new drugs were developed with the aim of combining a rapid action, a tolerable profile, and higher effectiveness on specific symptoms, which were relatively poorly targeted by standard antidepressants, such as anhedonia and response to reward, suicidal ideation/behaviours, insomnia, cognitive deficits, and irritability. This review discusses the clinical specificity profile of new antidepressants, namely 4-chlorokynurenine (AV-101), dextromethorphan-bupropion, pregn-4-en-20-yn-3-one (PH-10), pimavanserin, PRAX-114, psilocybin, esmethadone (REL-1017/dextromethadone), seltorexant (JNJ-42847922/MIN-202), and zuranolone (SAGE-217). The main aim is to provide an overview of the efficacy/tolerability of these compounds in patients with mood disorders having different symptom/comorbidity patterns, to help clinicians in the optimization of the risk/benefit ratio when prescribing these drugs.

## Introduction

Globally, mood disorders are a severe and chronic cause of disability, morbidity, and mortality (James *et al.*, 2018). They impact social functioning, as well as work productivity, resulting in a significant socioeconomic burden (Egede *et al.*, 2016). Moreover, these disorders have a strong impact on physical health and significantly increase the risk of developing other medical conditions, such as heart disease, diabetes, and obesity (Fanelli and Serretti, 2022; Wimberley *et al.*, 2022). We cannot forget to mention one of the most urgent clinical concerns associated with depression, that is, suicide, as the pooled lifetime prevalence of suicide attempts is between 27 and 34% in people with depressive disorders (WHO depressive disorder, 2023). Antidepressant drugs, such as selective serotonin reuptake inhibitors, serotonin and norepinephrine reuptake inhibitors, and tricyclic antidepressants, are widely prescribed in the general population (Dörks *et al.*, 2022) and used to treat depressive symptoms, as well as anxiety and obsessive symptoms, among others (Castle *et al.*, 2021); however, they are associated with variable treatment outcomes that are difficult to predict (Fanelli *et al.*, 2022). Consequently, finding an effective treatment for all patients with major depressive disorder (MDD), bipolar depression, postpartum depression (PPD), perimenopausal depression (PMD), and depression associated with general medical conditions remains a major unmet need.

Treatment-resistant depression (TRD) is usually defined as an ineffective response to at least two antidepressant drugs of adequate dose and duration (Gaynes *et al.*, 2020), but also in patients who do achieve remission, residual symptoms are common (McClintock *et al.*, 2011). TRD is particularly common in patients suffering from bipolar depression. The high burden of depressive phases in bipolar disorder is partly linked to the fact that there is only a limited number of approved treatment options for bipolar depression, and in most cases the evidence is controversial (McIntyre and Calabrese, 2019). Poor response to treatment (residual symptoms, slow improvement, or TRD) may be highly burdensome and associated with additional risks in some groups of patients, such as women in the peripartum. If not successfully treated, serious consequences can negatively affect the well being and health of both mother and child (Beck, 2006). More specifically, it increases the risk of preeclampsia, preterm delivery, maternal substance abuse, and suicidality (Vliegen *et al.*, 2014), as well as causing significant impairment in both maternal functioning and mother-infant attachment (Gagliardi *et al.*, 2012). This can have a negative impact on child cognitive, behavioural, and emotional development, with long-lasting repercussions (Frieder *et al.*, 2019).

Poor treatment response is not the only issue of drugs currently approved for depressive disorders. Adverse events are indeed quite common, and not infrequently they compromise treatment adherence (Hu *et al.*, 2004). Standard antidepressant treatments also show delayed action, as they require 4–6 weeks before reaching the complete therapeutic effects, and in the meantime, patients remain vulnerable to the risk of suicide (Machado-Vieira *et al.*, 2009). The discussed points emphasize the urgent need for more broadly effective and rapid interventions in depression, especially with a rapid antisuicidal and antianhedonic effect. Recent preclinical and clinical studies are testing new glutamate drugs, for example, acting as n-methyl-d-aspartate receptor (NMDAR) antagonists (Salituro *et al.*, 1994). Other new compounds target gamma-aminobutyric acid (GABA) neurotransmission, such as GABA_A_ positive allosteric modulators (PAMs) (Althaus *et al.*, 2020), and the orexin system, such as seltorexant (Recourt *et al.*, 2019). Psychedelic drugs such as psilocybin have also attracted renewed interest in treating resistant cases (Griffiths *et al.*, 2016), based on their effects on cognitive flexibility (Boulougouris *et al.*, 2008) and associative learning (Harvey, 2003). Despite new drugs having general mechanisms alternative to the ‘classic’ monoaminergic hypothesis, pimavanserin is a new compound studied for depression that principally acts on the well studied serotonergic system.

The purpose of this review is to summarize and discuss the available literature on novel rapid acting antidepressants, in various stages of development, with respect to their antidepressant efficacy, safety, and tolerability, in patients with different mood disorders, also considering each drug-specific pharmacodynamic profile.

## Search strategy and inclusion criteria

The search was conducted using the National Institutes of Health US National Library of Medicine Clinical Trials Database and PubMed, retrieving relevant data up to September 2022. The used keywords were: ‘av-101’, ‘dextromethorphan-bupropion’, ‘AXS-05’, ‘REL-1017’, ‘esmethadone’, ‘zuranolone’, ‘SAGE-17’, ‘PRAX-114’, ‘pimavanserin’, ‘psilocybin’, ‘seltorexant’, ‘MIN-202’, ‘PH-10’; each of these was combined with each of the following: ‘major depressive disorder’, ‘depression’, ‘antidepressant’, ‘depressive symptoms’, ‘bipolar depression’, ‘post-partum depression’. The drugs of interest were selected based on the availability of at least one clinical trial (any phase), completed or in progress, studying their possible efficacy in the treatment of depressive disorders. We did not include drugs already approved for the treatment of depressive disorders, except for dextromethorphan-bupropion (AXS-05) which was recently approved by the Food and Drug Administration (FDA) and can be still considered a novel rapid acting antidepressant. We did not consider drugs with insufficient evidence of efficacy on specific depressive symptoms, or without an innovative mechanism that would suggest a potential effectiveness.

The eligibility criteria were: published (including congress abstracts) or unpublished clinical trials (relevant information was also extracted from reviews, meta-analysis, and websites of biopharmaceutical companies); focused on the clinical effects on the mentioned new compounds in patients with depressive disorders as main diagnosis or comorbidity, or in healthy volunteers; written in English. This is a narrative review, therefore a quantitative synthesis and systematic evaluation of the quality of the included studies are not among the scope of this work.

## Results

**Fig. 1:**
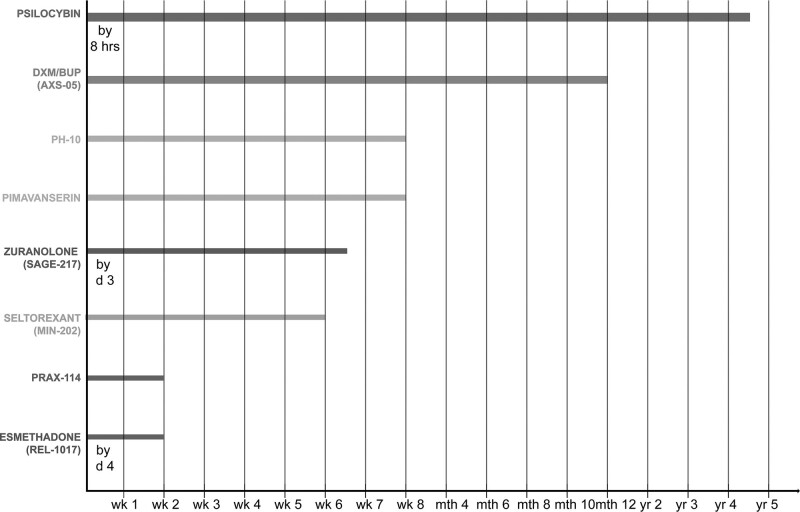
Earliest time of onset (indicated as the week of initial antidepressant effect) and longest duration of antidepressant efficacy (indicated as the longest period, that is, weeks, months, or years, of sustained antidepressant effect) reported for the included drugs in at least study. For psilocybin, zuranolone, and esmethadone, data are available on the onset of therapeutic effect before the first week of treatment. For AXS-05, PH-10, pimavanserin, seltorexant (MIN-202), and PRAX-114 there are no data about a possible early onset of the therapeutic effect (i.e. during the first hours or first days of treatment). BUP, bupropion; d, day; DXM, dextromethorphan; hr, hour; mth, month; wk, week; yr, year.

The main characteristics and studied indications of the nine compounds included in our review are presented in Table 1. They have been studied mostly for the treatment of MDD, as adjunctive treatment or monotherapy, often for TRD cases. Despite this leaving a gap regarding their potential benefits in other depressive disorders, the focus of this review is on the efficacy profile in terms of specific symptoms with cross-diagnostic relevance, rather than for specific categorical diagnoses. As one of the key parameters for the evaluation of new compounds is the time of antidepressant effect onset, we represented this in Fig. 1, together with the longest observed period in which the antidepressant effect was sustained. Completed and published clinical studies are summarized in Table 2. Completed clinical studies but not published in peer-reviewed journals are shown in Table 3 and incomplete studies until September 2022 are presented in Table 4.

**Table 1:** Characteristics and studied indications of the included novel rapid acting antidepressant drugs according to the results of at least one study considered

Drug	Mechanism of action/pharmacology	Route of administration	Recommended dose per day	Possible condition of effectiveness in human samples
4-Chlorokynurenine (AV-101)	Competitive NMDAR antagonism	Orally	1080 or 1440 mg	None
Esmethadone (REL-1017)	Noncompetitive NMDAR antagonism	Orally	25 or 50 mg	Adjunctive treatment to standard antidepressants in MDD
Dextromethorphan-bupropion (AXS-05)	Noncompetitive NMDAR antagonism	Orally	45 mg dextromethorphan/105 mg bupropion	Monotherapy in MDD and TRD
Psilocybin	5-HT2_A_R agonism	Orally	10 or 20 or 25 or 30 mg	Monotherapy in MDD and TRD monotherapy in cancer and AIDS-related depression and anxiety (PAP)
Pimavanserin	5-HT2_A_R antagonism/inverse agonism	Orally	34 mg	Adjunctive treatment to standard antidepressants in MDD monotherapy and adjunctive treatment to standard antidepressants in MDD associated with Parkinson’s disease
Zuranolone (SAGE-217)	GABA_A_R PAM	Orally	30 or 50 mg	Monotherapy and adjunctive treatment to standard antidepressants in MDD monotherapy in PPD monotherapy in bipolar depression
Prax-114	GABA_A_R PAM	Orally	10 or 20 or 40 or 60 mg	Monotherapy in PMD
Ph-10 (pregn-4-en-20-yn-3-one)	Nasal chemosensory receptor modulator	Intranasally	3.2 or 6.4 µg	Monotherapy in MDD
Seltorexant (JNJ-42847922/MIN-202)	Selective OX-2R antagonism	Orally	10 or 20 or 40 mg	Adjunctive treatment to standard antidepressants in MDD

GABA_A_R, gamma-aminobutyric acid type _A_ receptor; MDD, major depressive disorder; NMDAR, n-methyl-d-aspartate receptor; OX-2R, orexin type 2 receptor; PAM, positive allosteric modulator; PAP, psilocybin assisted psychotherapy; PMD, perimenopausal depression; PPD, postpartum depression; R, receptor; TRD, treatment-resistant depression.

**Table 2:** Completed clinical trials until September 2022 and published in peer-reviewed journals

Mood disorder	Drug	Dose	Phase	Type of study	Duration^a^	Follow up^b^	NCT number	Trial design	Adjunctive treatment or monotherapy	Depressive symptoms severty scale at baseline	Population			Drug effectiveness on depressive symptoms
											*N*	Sex (F/M)	Mean age or range	
MDD	REL-1017	25–50 mg	II	Multicenter randomized double-blind placebo-controlled trial	1 week	15–21 days	NCT03051256	3 arms REL-1017 50 (*n* 21) REL-1017 25 (*n* 19) Placebo (*n* 22)	Adjunctive treatment	MADRS 33.8	62	45.2%/54.8%	49.2	Reduction in depressive symptoms at week 1 MADRS: −17.4 for REL-1017 25 [*P* = 0.0122] [*d* = 0.8] vs −15.9 for REL-1017 50 [*P* = 0.0308] [*d* = 0.7] vs −8 for placebo (Primary outcome)
	AXS-05	45/105 mg	II	Multicenter randomized double-blind parallel-group trial	6 weeks	At week 7	NCT03595579	2 arms AXS-05 (*n* 43) Bupropion (*n* 37)	Monotherapy	MADRS 31.8	97	64%/38.1%	37.5	Reduction in depressive symptoms at week 6 MADRS: −17.3 for AXS-05 vs -12.1 for BUP [*P* = 0.013] [d = 0.5] (Primary outcome)
		45/105 mg	III	Multicenter randomized double-blind placebo-controlled trial	6 weeks	At week 7	NCT04019704	2 arms AXS-05 (*n* 156) Placebo (*n* 162)	Monotherapy	MADRS 33	327	66%/34%	42	Reduction in depressive symptoms at week 6 MADRS: −16.6 for AXS-05 vs −11.9 for placebo [*P* = 0.002][*d* = 0.38] (Primary outcome)
	Psilocybin	25 mg 1 mg	II	Randomized double-blind controlled trial	6 weeks	6 months	NCT03429075	2 arms Psilocybin + placebo (*n* 30) Psilocybin + escitalopram (*n* 29)	Adjunctive treatment and monotherapy (PAP)	QIDS-SR-16 14.5	59	33.8%/66.1%	41.2	No difference in antidepressant effect between psilocybin and escitalopram at week 6 QIDS-SR-16: −8.0 for psilocybin vs −6.0 for escitalopram [*P* = 0.17] (Primary outcome)
		20 mg/70 kg 30 mg/70 kg	II	Randomized waiting-list controlled trial	8 weeks (immediate treatment) 13 weeks (delayed treatment)	4 weeks	NCT03181529	2 arms Immediate treatment (*n* 13) Delayed treatment (*n* 11)	Monotherapy (PAP)	GRID-HDRS 22.8	27	67%/33%	39.8	Reduction in depressive symptoms GRID-HDRS: 8.0 at week 5 [d = 2.5], 8.5 at week 8 [2.6] for immediate treatment group [*P* < 0,001] GRID- HDRS: 23.8 at week 5 [*d* = 2.5], 23.5 at week 8 [2.6] for delayed treatment group [*P* < 0.001] (Primary outcome)
		25 mg	I	Randomized double-blind placebo-controlled crossover trial	2 weeks	0	NCT03912974	2 arms Escitalopram + psilocybin Placebo + psilocybin	Monotherapy (PAP)	Healthy subjects	27	47.9%/52.1%	34	Escitalopram pretreatment reduced psilocybin adverse effects [*P* = 0.004], fear [*P* = 0.004], anxiety [*P* < 0.05], adverse autonomic effects [*P* < 0.02] (Primary outcome)
	Pimavanserin	34 mg	II	Randomized double-blind placebo-controlled trial	10 weeks	4 weeks	NCT03018340	2 arms Pimavanserin (*n* 52) Placebo (*n* 155)	Adjunctive treatment	MADRS > 20	207	72.9%/27.1%	≥ 18	Reduction in depressive symptoms at week 5 HDRS-17: −11.5 for pimavanserin vs −7.5 for placebo [*P* = 0.003] [*d* = 0.63] (Primary outcome)
	Zuranolone	30 mg	II	Randomized double-blind placebo-controlled trial	2 weeks	6 weeks	NCT03000530	2 arms Zuranolone (*n* 45) Placebo (*n* 44)	Monotherapy	HDRS-17 25.2	89	62.7%/37.3%	44.3	Reduction in depressive symptoms at day 15 HDRS-17: −17.4 for zuranolone vs −10.3 for placebo [*P* = 0.0005] [*d* = 81]
	Ph-10	3.2 µg 6.4 µg	NA	Randomized double-blind placebo-controlled parallel-group trial	8 weeks	1 week	ISSN2456-9836	3 arms PH-10 6. PH-10 3.2 Placebo	Monotherapy	HDRS-17 PH10 6.4 24.7 HDRS-17 PH10 3.2 22.4	30	60%/40%	PH10 6.4 46.6 PH10 3.2 33.2	Reduction in depressive symptoms at week 8 HDRS-17: −17.80 for PH-10 6.4 [*P* = 0.022] [*d* = 0.95] vs −16.3 for PH-10 3.2 [*P* = 0.101] [*d* = 0.74] vs −10.9 for placebo (Primary outcome)
	Seltorexant	20 mg	I	Multicenter randomized double-blind placebo-controlled parallel-group trial	10 days or 28 days	2 weeks	NCT02476058	3 arms Seltorexant (*n* 22) Placebo (*n* 12) Active placebo (*n* 13)	Monotherapy and adjunctive treatment	IDS-C30 36.8	47	34%/66%	18–64	Reduction in depressive symptoms at day 11 HDRS-17: −5.5 for seltorexant vs −3.6 for placebo vs −4.1 for active placebo [*P* < 0.05] (Primary outcome)
		10 mg 20 mg 40 mg	I	Double-blind placebo-controlled four-way crossover trial	Single dose	1 week	NCT02067299	4 arms Placebo-seltorexant 10-20-40 Seltorexant 10-40-placebo-20 Seltorexant 20-placebo-40-10 Seltorexant 40-20-10-placebo	Adjunctive treatment	HDRS-17 9.35	20	60%/40%	43	Failed to improve depressive symptoms at week 1 QIDS-SR: −2.1 for Seltorexant 40 vs −0.7 for placebo
		10 mg 20 mg 40 mg	II	Multicenter randomized double-blind placebo-controlled dose-finding trial	6 weeks	2 weeks	NCT03227224	4 arms: Seltorexant 10 (*n* 33) Seltorexant 20 (*n* 61) Seltorexant 40 (*n* 52) Placebo (*n* 137)	Adjunctive treatment	MADRS ≥ 25	287	53.7%/46.3%	49.1	Reduction in depressive symptoms at week 6 MADRS: −3.1 for SLX 20 vs -1.5 for SLX 40 [*P* = 0.083] (Primary outcome)
TRD	AV-101	1080 mg 1440 mg	II	Randomized double-blind placebo-controlled crossover Trial	2 weeks	0	NCT02484456	2 arms AV-101-placebo Placebo-AV-101	Monotherapy	HDRS ≥ 18	19	47.4%/52.6%	41.28	Failed to improve depressive symptoms at day 15 in the HDRS [*P* = 0.16] [*d* = 0.22] (Primary outcome)
		720 mg 1440 mg	I	Randomized double-blind placebo-controlled crossover trial	3 weeks	0	NCT03583554	3 arms Placebo-AV-101 720- 1440 AV-101 720-1440-placebo AV-101 1440-placebo 720	Monotherapy	Healthy subjects	10	8.3%/91.7%	32.6	1440 mg showed consistent NMDAR blockade (Primary outcome)
	Psilocybin	10 mg 25 mg	NA	Open-label trial	2 psilocybin sessions 1 week apart	3 months	ISRCTN14426797	Single arm	Monotherapy (PAP)	QIDS 19.2	12	50%/50%	44.7	Reduction in depressive symptoms at 1 week and month 3 QIDS psilocybin 25: −11.8 at week 1 [*P* = 0.002] [g = 3.1] vs −9.2 at month 3 [*P* = 0.003] [g = 2] (Primary outcome)
PPD	Zuranolone	30 mg	III	Randomized double-blind placebo-controlled trial	2 weeks	4 weeks	NCT02978326	2 arms Zuranolone (*n* 76) Placebo (*n* 74)	Monotherapy	HDRS-17 ≥ 28.4	153	100%/0%	28.3	Reduction in depressive symptoms at day 15 HDRS-17: -17.8 for ZRN vs -13.6 for placebo [*P* = 0.003] [d = 0.53] (Primary outcome)
MDD and Parkinson’s disease	Pimavanserin	34 mg	II	Open-label trial	8 weeks	2 weeks	NCT03482882	Single arm	Monotherapy (*n* 21) adjunctive treatment (*n* 24)	HDRS 19.2	45	48.9%/51.1%	69.3	Reduction in depressive symptoms at week 8 HDRS-17: -11.2 for monotherapy vs -10.2 for adjunctive therapy [*P* < 0.0001]
Cancer-related depression and anxiety	Psilocybin	0.2 mg/kg	I/II	Randomized double-blind placebo-controlled trial	2 psilocybin sessions, several weeks apart	6 months	NCT00302744	2 arms Psilocybin Active placebo	Monotherapy (PAP)	BDI ≥ 15	12	91.7%/8.3%	36–58	Failed to improve depressive symptoms (BDI) and anxiety symptoms (STAI) at week 2 (Primary outcome) STAI reduced at month 1 [*P* = 0.001] and at month 3 [*P* = 0.03] BDI reduced at month 6 [*P* = 0.03]
		1 or 3 mg/70 kg 22 or 30 mg/70 kg	II	Randomized double-blind crossover trial	2 psilocybin sessions, 5 weeks apart	6 months	NCT00465595	2 arms Psilocybin 1 or 3 first (*n* 25) Psilocybin 22 or 30 first (*n* 26)	Monotherapy (PAP)	GRID-HDRS-17 22	56	49%/51%	56.3	Reduction in depressive symptoms at 6 months GRID-HDRS-17: clinical response rate of 78% for two-dose sequence group [*d* = 1.55] (Primary outcome) Reduction in anxiety symptoms at 6 months HAM-A: clinical response rate of 83% for two-dose sequence group [d = 1.55] (Primary outcome)
		0.3 mg/kg	I	Randomized double-blind placebo-controlled crossover Trial	2 psilocybin sessions, 7 weeks apart	6 months	NCT00957359	2 arms Psilocybin first (*n* 14) Active placebo first (*n* 15)	Monotherapy (PAP)	BDI ≥ 15	31	62.1%/37.9%	56.28	Reduction in depressive symptoms at week 7 (prior the crossover) BDI: clinical response rate of 83% for psilocybin group first vs 14% for active placebo group first [*P* < 0.05][*d* = 0.82] (Primary outcome) Reduction in anxiety symptoms at week 7 (prior the crossover) HADS: clinical response rate of 58% for PSY group first vs 14% for active placebo group first [*P* ≤ 0.01][d = 1.07] (Primary outcome)
		0.3 mg/kg	NA	Randomized double-blind placebo-controlled crossover trial	2 psilocybin sessions, 7 weeks apart	4.5 years	NA	2 arms Psilocybin first (*n* 6) Active placebo first (*n* 5)	Monotherapy (PAP)	NA	11	63.6%/36.4%	60.3	Reduction in suicidal ideation at 8 h [*P* < 0.001] and sustained for 6.5 months [*P* < 0.001] (Primary outcome) Reduction in LoM at 2 weeks [*P* = 0.005] and sustained for 6.5 months [*P* < 0.001], 3.2 years [*P* < 0.001], and 4.5 years [*P* < 0.001]
AIDS-related depression	Psilocybin	0.3 mg/kg 0.36 mg/kg	I	Open-label trial	1 psilocybin session	3 months	NCT02950467	Single arm	Monotherapy (PAP)	DS-II ≥ 8/32	18	0%/100%	59.2	Reduction in demoralization at week 3 DS-II: −5.78 [hp2 = 0.47] (Primary outcome)

For some studies, some data is not available (i.e. phase study, depressive symptoms severity scale at baseline, *P* value, effect size).

BDI, Beck Depression Inventory total score; DS-II, Demoralization Scale-II total score; GRID-HDRS, Grid-Hamilton Depression Rating Scale total score; HADS, Hospital Anxiety and Depression Scale total score; HAM-A, Hamilton Anxiety Rating Scale total score; HDRS, Hamilton Depression Rating Scale total score; IDS-C, Inventory of Depressive Symptomatology Clinician Rating total score; LoM, loss of meaning; MADRS, Montgomery-Asberg Depression Rating Scale total score; MDD, major depressive disorder; NA, not available; PAP, psilocybin assisted psychotherapy; PPD, postpartum depression; QIDS, Quick Inventory of Depressive Symptomatology total score; QIDS-SR, Quick Inventory of Depressive Symptomatology-Self Reported total score; STAI, State-Trait Anxiety Inventory total score; TRD, treatment-resistant depression.

a Refers to the length over time of the pharmacological intervention.

b Refers to the length of monitoring over time of participant’s health after the end of the pharmacological intervention.

**Table 3:** Completed clinical trials but not published in peer-reviewed journals until September 2022

Mood disorder	Drug	Dose	Phase	Type of study	Duration^a^	Follow up^b^	NCT number	Trial design	Adjunctive treatment or monotherapy	Depressive symptoms severity scale at baseline	Population			Drug effectiveness on depressive symptoms
											*N*	Sex (F/M)	Age range or mean age	
MDD	AV-101	1440 mg	II	Multicenter randomized double-blind placebo-controlled parallel-group Trial	2 weeks	NA	NCT03078322	2 arms AV-101 Placebo	Adjunctive treatment	HDRS-17 > 20	199	NA	18–65	Failed to improve depressive symptoms at day 15 in the MADRS (Primary outcome)
	AXS-05	45/105 mg	III	Multicenter open-label trial	12 months	NA	NCT04039022	Single arm	Monotherapy	MADRS 33	876	62%/38%	42.4	Reduction in depressive symptoms at week 6 MADRS: −21.1 (Primary outcome)
		45/105 mg	II	Multicenter open-label trial	12 months	NA	Substudy of NCT04039022 (antidepressant unresponsive patients)	Single arm	Monotherapy	MADRS 33.3	115	NA	NA	Reduction in depressive symptoms at week 6 MADRS: −19.1 (Primary outcome)
		45/105 mg	III	Multicenter open-label trial	12 months	NA	Substudy of NCT04039022 (patients with suicidal ideation)	Single arm	Monotherapy	MADRS-suicidal ideation 3.4 MADRS 36.8	37	NA	NA	Reduction in suicidal ideation at week 3 MADRS-SI: -2.8 [*P* = 0,001] (Primary outcome)
	Pimavanserin	34 mg	III	Multicenter randomized double-blind placebo-controlled parallel-group trials	6 weeks	NA	NCT03968159 and NCT03999918	2 arms Pimavanserin (*n* 148) Placebo (*n* 150)	Adjunctive treatment	NA	298	69.8%/30.2%	≥ 18	Failed to improve depressive symptoms at week 5 HDRS-17: −9.0 for pimavanserin vs −8.1 for placebo [*P* = 0.296] (Primary outcome)
	Zuranolone	50 mg	III	Multicenter randomized double-blind placebo-controlled trial	2 weeks	Day 42	NCT04442490	2 arms Zuranolone (*n* 27) Placebo (*n* 272)	Monotherapy	HDRS-17 26.8	543	66%/34%	39.7	Reduction in depressive symptoms at day 15 HDRS-17: −14.1 for ZRN vs −12.3 for placebo [*P* = 0.0141] (Primary outcome)
		30 mg 50 mg	III	Open-label trial	2 weeks	1 year	NCT03864614	Single arm	Monotherapy (*n* 421) Adjunctive treatment (*n* 304)	HDRS-17 25.3	725	NA	18–75	Reduction in depressive symptoms at day 15 HDRS-17: −14.9 for zuranolone 30 vs −15.9 for zuranolone 50
		50 mg	III	Randomized double-blind placebo-controlled trial	2 weeks	6 weeks	NCT04476030	2 arms Zuranolone (*n* 210) Placebo (*n* 215)	Adjunctive treatment	HDRS-17 26.8	440	NA	18–64	Reduction in depressive symptoms at day 3 HDRS-17: −8.9 for zuranolone vs −7.0 for placebo [*P* = 0.0004] (Primary outcome)
		20 mg 30 mg	III	Multicenter randomized double-blind placebo-controlled trial	2 weeks	Days 43–182	NCT03672175	3 arms Zuranolone 20 (*n* 159) Zuranolone 30 (*n* 166) Placebo (*n* 157)	Monotherapy	HDRS-17 ≥ 22	581	70.3%/29.7%	18–65	Failed to improve depressive symptoms at day 15 HDRS-17: –12.5 for zuranolone 30 vs -11.1 for placebo [*P* = 0.116] [*d* = 0.17] vs –11.5 for zuranolone 20 [*P* = 0.664] [*d* = 0.03] (Primary outcome)
	Prax-114	40 mg	II/III	Randomized double-blind placebo-controlled trial	4 weeks	2 weeks	NCT04832425	2 arms PRAX-114 Placebo	Monotherapy	HDRS-17 ≥ 23	216	NA	18–65	Failed to improve depressive symptoms at day 15 in the HDRS-17 (Primary outcome)
	Seltorexant	20 mg 40 mg	II	Multicenter randomized double-blind flexible-dose parallel-group trial	6 months	2 weeks	NCT03321526	2 arms Seltorexant (*n* 52) Quetiapine (extended-release) (*n* 52)	Adjunctive treatment	NA	104	66.3%/33.7%	18–84	Failed to improve depressive symptoms at week 12 in the MADRS
TRD	AXS-05	45/105 mg	II	Multicenter randomized double-blind placebo-controlled trial	52 weeks	0	NCT04608396	2 arms AXS-05 (*n* 22) Placebo (*n* 22)	Monotherapy	NA	44	NA	≥ 18	Delayed the time to relapse of depressive symptoms up to 52 weeks [*P* = 0.002] (Primary outcome)
		45/105 mg	III	Randomized double-blind active-controlled two-period trial	12 weeks	0	NCT02741791	2 arms AXS-05 (*n* 156) Bupropion (*n* 156)	Monotherapy	NA	312	NA	18–65	Failed to improve depressive symptoms at week 6 in the MADRS [*P* = 0.117] [*d* = 0.21] (Primary outcome)
		45/105 mg	II	Multicenter open-label trial	12 months	0	NCT04634669	Single arm	Monotherapy	MADRS 32.2	150	60.7%/39.3%	45.6	Reduction in depressive symptoms at 12 months MADRS: −24.5 [*P* < 0.001] (Primary outcome)
	Psilocybin	1 mg 10 mg 25 mg	II	Randomized double-blind controlled trial	Single treatment session	12 weeks	NCT03775200	3 arms Psilocybin 1 Psilocybin 10 Psilocybin 25	Monotherapy (PAP)	NA	233	NA	≥18	Reduction in depressive symptoms at week 3 MADRS: −6.6 Psilocybin 25 vs psilocybin 1 [*P* < 0.001] (Primary outcome)
PPD	Zuranolone	50 mg	III	Randomized double-blind placebo-controlled trial	2 weeks	4 weeks	NCT04442503	2 arms Zuranolone (*n* 97) Placebo (*n* 98)	Monotherapy	HDRS-17 ≥ 26	195	NA	18–45	Reduction in depressive symptoms at day 15 HDRS-17: −15.6 for zuranolone vs −11.6 for placebo [*P* = 0.0007] (Primary outcome)
Bipolar depression	Zuranolone	30 mg	III	Open-label trial	2 weeks	4 weeks	NCT03692910	2 arms Zuranolone Placebo	Monotherapy	HDRS 25.7	35	23%/12%	47.6	Reduction in depressive symptoms MADRS: −7.7 on day 3, -15.5 on day 15, -16.4 on day 42
PMD	Prax-114	60 mg	II	Open-label trial	2 weeks	2 weeks	NA	Single arm	Monotherapy	HDRS-17 25.3	6	100%/0%	NA	Reduction in depressive symptoms at day 15 HDRS-17: −12 (Primary outcome)

For some studies, some data is not available (i.e. follow-up, depressive symptoms severity scale at baseline, sex, age range or mean age, *P* value, effect size).

HDRS, Hamilton Depression Rating Scale total score; MADRS, Montgomery-Asberg Depression Rating Scale total score; MADRS-SI, Montgomery-Asberg Depression Rating Scale-Suicidal Ideation total score; MDD, major depressive disorder; NA, not available; PAP, psilocybin assisted psychotherapy; PMD, perimenopausal depression; PPD, postpartum depression; TRD, treatment-resistant depression.

a Refers to the length over time of the pharmacological intervention.

b Refers to the length of monitoring over time of participant’s health after the end of the pharmacological intervention.

**Table 4:** Ongoing, terminated, or completed clinical trials without results posted until September 2022

Mood disorder	Drug	Dose	Phase	Type of study	NCT number	Trial design	Adjunctive treatment or monotherapy	Depressive symptoms severity scale at baseline	Population		Drug effectiveness on depressive symptoms	Status
									*N*	Range age		
MDD	REL-1017	25 mg	III	Multicenter randomized double-blind placebo-controlled trial	NCT04688164	2 arms REL-1017 Placebo	Adjunctive treatment	NA	400	18–65	Reduction in depressive symptoms at day 28 in the MADRS (Primary outcome)	No results posted
		25 mg	III	Multicenter randomized double-blind placebo-controlled trial	NCT04855747	2 arms REL-1017 Placebo	Adjunctive treatment	NA	400	18–65	Reduction in depressive symptoms at day 28 in the MADRS (Primary outcome)	Recruiting
		25 mg	III	Multicenter randomized double-blind placebo-controlled trial	NCT05081167	2 arms REL-1017 Placebo	Monotherapy	NA	400	18–65	Reduction in depressive symptoms at day 28 in the MADRS (Primary outcome)	No results posted
		25 mg	III	Open-label trial	NCT04855760	Single arm	Adjunctive treatment	NA	600	18–65	Incidence of TEAEs (Primary outcome)	Recruiting
	Psilocybin	25 mg	II	Randomized double-blind support-of-concept trial	NCT03866174	2 arms Psilocybin Active placebo	Monotherapy (PAP)	NA	100	21–65	Reduction in depressive symptoms at day 43 in the MADRS (Primary outcome)	Not recruiting
		0.215 mg/kg	II	Randomized double-blind placebo-controlled trial	NCT03715127	2 arms Psilocybin Placebo	Monotherapy (PAP)	MADRS ≥ 10/≤40	55	18–60	Reduction in depressive symptoms at day 32 in the MADRS and BDI (Primary outcome)	No results posted
		0.1 mg/kg 0.3 mg/kg	I	Randomized double-blind placebo-controlled crossover trial	NCT03554174	4 arms Placebo-psilocybin 0.1 Placebo-psilocybin 0.3 Psilocybin 0.1-placebo Psilocybin 0.3-placebo	Monotherapy (PAP)	NA	18	18–65	Reduction in depressive symptoms at weeks 1 and 2 after each experimental session in the GRID-HDRS	Not recruiting
		25 mg	II	Randomized double-blind placebo-controlled trial	NCT03380442	3 arms Psilocybin Ketamine No treatment group	Monotherapy (PAP)	HDRS ≥ 17	60	18–64	Reduction in depressive symptoms at months 3 and 6 in the QIDS (Primary outcome)	Unknown
		25 mg	II	Randomized double-blind placebo-controlled trial	NCT04620759	2 arms Psilocybin Placebo	Monotherapy (PAP)	GRID-HDRS ≥ 18	90	21–65	Reduction in depressive symptoms at month 1 in the GRID-HDRS (Primary outcome)	Recruiting
		25 mg	II	Randomized double-blind placebo-controlled trial	NCT04630964	2 arms Psilocybin Active placebo	Monotherapy (PAP)	MADRS > 22/≤30	35	20–65	Reduction in depressive symptoms at day 8 in the MADRS (Primary outcome)	Not recruiting
	Pimavanserin	34 mg	III	Open-label trial	NCT04000009	Single arm	Monotherapy	NA	235	>18	Number of participants with TEAEs (Primary outcome)	Terminated for business reasons and not due to safety concerns
	Zuranolone	30 mg	III	Randomized double-blind placebo-controlled trial	NCT03771664	2 arms Zuranolone Placebo	Monotherapy	HDRS ≥ 20	87	18–64	Improvement of sleep efficiency assessed by polysomnography on day 14 (Primary outcome)	Terminated (internal company decisions)
		30 mg	III	Randomized double-blind placebo-controlled trial	NCT04007367	2 arms Zuranolone Placebo	Monotherapy	HDRS ≥ 20	52	18–65	Time to relapse of depressive symptoms (Primary outcome)	Terminated (internal company decisions)
	PRAX-114	10 mg 20 mg 40 mg 60 mg	II	Randomized double-blind placebo-controlled dose-ranging trial	NCT04969510	5 arms PRAX-114 10 PRAX-114 20 PRAX-114 40 PRAX-114 60 Placebo	Monotherapy adjunctive treatment	HDRS-17 ≥ 23	110	18–65	Reduction in depressive symptoms at day 15 in the HDRS-17 (Primary outcome)	No results posted
	Seltorexant	NA	I	Randomized double-blind placebo-controlled trial	NCT04951609	2 arms Seltorexant Placebo	Adjunctive treatment	NA	52	12–17	Reduction in depressive symptoms at week 6 in the MADRS	Recruiting
		NA	I	Multicenter randomized double-blind Placebo and positive controlled four-way crossover Trial	NCT04451187	4 arms Seltorexant dose 1 Seltorexant dose 2 Placebo Zoplicone	Adjunctive treatment	MADRS ≥ 18	63	21–80	Driving performance as assessed in an on-road driving test (Primary outcome)	Not recruiting
		NA	III	Multicenter randomized double-blind placebo-controlled parallel-group trial	NCT04532749	2 arms Seltorexant Placebo	Adjunctive treatment	HDRS-17 ≥ 20	212	18–74	Reduction in depressive symptoms at day 43 in the MADRS (Primary outcome)	Stopped as a result of the interim analysis -no results posted
		NA	III	Multicenter randomized double-blind placebo-controlled parallel-group trial	NCT04533529	2 arms Seltorexant Placebo	Adjunctive treatment	HDRS-17 ≥ 20	550	18–74	Reduction in depressive symptoms at week 6 in the MADRS (Primary outcome)	Recruiting
		NA	III	Randomized double-blind parallel-group trial	NCT04513912	2 arms Seltorexant quetiapine (extended-release)	Adjunctive treatment	HDRS-17 ≥ 20	720	18–74	Treatment response at week 26 in the MADRS (Primary outcome)	Recruiting
TRD	AXS-05	45/105 mg	II	Randomized double-blind active placebo-controlled Trial	NCT04971291	2 arms AXS-05 Bupropion	Monotherapy	NA	312	18–65	Reduction in depressive symptoms at week 6 in the MADRS (Primary outcome)	Enrolling by invitation
	Psilocybin	5 mg 25 mg	II	Randomized double-blind active placebo-controlled parallel-group trial	NCT04670081	3 arms Psilocybin 5 Psilocybin 25 Placebo	Monotherapy (PAP)	NA	144	25–65	Treatment response at week 6 in the HDRS (Primary outcome)	Recruiting

For some studies, some data is not available (i.e. drug’s dose and depressive symptoms severity scale at baseline).

BDI, Beck Depression Inventory total score; GRID-HDRS, Grid-Hamilton Depression Rating Scale total score; HDRS, Hamilton Depression Rating Scale total score; MADRS, Montgomery-Asberg Depression Rating Scale total score; MDD, major depressive disorder; NA, not available; NMDAR, n-methyl-d-aspartate receptor; PAP, psilocybin assisted psychotherapy; QIDS, Quick Inventory of Depressive Symptomatology total score; TEAE, treatment-emergent adverse event; TRD, treatment-resistant depression.

### Compounds acting on the glutamatergic system

#### 4-Chlorokynurenine

Av-101 (4-chlorokyneurine, 4-Cl-KYN) is a prodrug, rapidly converted to 7-chloro-kynurenic acid (7-Cl-KYNA) by kynurenine aminotransferase (Salituro *et al.*, 1994). 7-Cl-KYNA exerts a neuroprotective action by a full antagonism activity on the glycine B co-agonist site of the NMDAR (Zanos *et al.*, 2015). The effects of this drug are also linked to the metabolite 4-chloro-3-hydroxyanthranilic acid (4-Cl-3-HAA), which inhibits the quinolinic acid, a neurotoxic NMDAR agonist (Walsh *et al.*, 1994). Considering the evidence implicating the kynurenine pathway in the aetiology of mood disorders (Brown *et al.*, 2021), and the rapid, dose-dependent, and sustained antidepressant effect following a single administration in an animal study (Zanos *et al.*, 2015), AV-101 was tested in humans; however, it failed to demonstrate benefits on depressive symptoms at week 2, according to two randomized clinical trials (RCTs) in patients with MDD (ClinicalTrials.gov Identifier NCT03078322 and NCT02484456). The drug failed both as adjunctive treatment to standard antidepressants (1440 mg/day for 2 weeks) (VistaGen Therapeutics, 2019) and in TRD as monotherapy (1080 mg/day for the first week, then 1440 mg/day for the second week) (Park *et al.*, 2020). These negative results raised the question of whether AV-101 could penetrate the blood-brain barrier. To address this question, dose-related effects of AV-101 (720 and 1440 mg) on NMDARs engagement were examined in a small sample of healthy individuals (ClinicalTrials.gov Identifier NCT03583554) (Murphy *et al.*, 2021). The dose of 1440 mg, but not 720 mg, showed consistent NMDAR blockade measured by an increase in electroencephalogram γ-band activity. Therefore, the dose of 1440 mg shows antagonist activity on the NMDARs, but no antidepressant effect was demonstrated in the available RCTs, which were both short-term studies.

#### Esmethadone

Rel-1017 is a dextro-isomer of racemic methadone (esmethadone or dextromethadone), with a low affinity and noncompetitive NMDARs antagonism profile (Bernstein *et al.*, 2019); it also shows a micromolar affinity for the serotonin transporter (SERT) and norepinephrine transporter (NET) (Nemeroff, 2022). Compared to its stereoisomer (levomethadone), it shows a lower affinity for opioid receptors and therefore a lack of opioid-like effects such as analgesic, tolerance and dependence properties, or sedation/respiratory depression (Gorman *et al.*, 1997). After demonstrating antidepressant activity in animal models, with modulation of synaptic connections and plasticity in the medial prefrontal cortex (Fogaça *et al.*, 2019), REL-1017 was tested in phase I clinical trials. In two multiple doses ascending studies, single doses of up to 150 mg and repeated dosing of up to 75 mg daily for 10 days exhibited linear pharmacokinetics and a good safety profile, characterized by dose-related nausea and somnolence at higher doses (Bernstein *et al.*, 2019). Rel-1017 received the FDA fast-track designation as an adjunctive treatment for MDD. There are two ongoing RCTs assessing the efficacy of this drug in MDD as an adjunctive treatment to the ongoing antidepressant in the short term (28 days) (ClinicalTrials.gov Identifier NCT04688164 and NCT04855747), and one well powered open-label study in the long term (1 year) (ClinicalTrials.gov Identifier NCT04855760). Other RCTs will consider REL-1017 as monotherapy (ClinicalTrials.gov Identifier NCT05081167) (Hecking *et al.*, 2021). While these studies are still ongoing and do not have results, a 1-week small pilot study reported promising findings (ClinicalTrials.gov Identifier NCT03051256). In this study, REL-1017 showed a rapid antidepressant effect (by day 4), sustained up to 7 days after the last dose, in patients with MDD maintained on their previous antidepressant regimen (to which they have had an inadequate response). Both 25 and 50 mg showed mood improvement associated with an enhancement in cognitive and social functioning, anxiety, agitation, anger, irritability, and sleep quality (Fava *et al.*, 2022).

#### Dextromethorphan-bupropion

AXS-05 is an oral combination of dextromethorphan and bupropion (Stahl, 2019). Dextromethorphan integrates the uncompetitive antagonism towards NMDARs with the sigma-1 receptor (S1R) agonism, the nicotinic acetylcholine receptors antagonism, and with activities on the SERT and NET (Taylor *et al.*, 2016). Bupropion primarily acts selectively blocking neuronal reuptake of noradrenaline and dopamine (Stahl, 2019). Additionally, bupropion prevents dextromethorphan from being rapidly metabolized by cytochrome P450 2D6 (CYP2D6), leading to an increase in its bioavailability (Hecking *et al.*, 2021). This innovative combination suggests a powerful pharmacological synergy and wide clinical use of AXS-05 across various psychiatric conditions (Stahl, 2019). The effectiveness of AXS-05 was proved with positive results in well powered studies of moderate or severe MDD, also in comorbidity with anxiety disorders. When compared with bupropion (Tabuteau *et al.*, 2022) or placebo (Iosifescu *et al.*, 2022), it had a more rapid onset of action (by week 1) and durable effect in the short-medium term (by week 6) in two phases II large RCTs (ClinicalTrials.gov Identifier NCT03595579 and NCT04019704), leading to FDA approval for MDD in August 2022. One open-label study (ClinicalTrials.gov Identifier NCT04039022) confirmed these promising findings in the long term (up to 12 months), showing an improvement in work/study, social and family functioning as well (Mardi, 2022), also in a sample unresponsive to other antidepressants (Axsome Therapeutics, 2020a). A very small study in a group of patients with suicidal ideation found a rapid remission of suicidal ideation by week 1, as well as an improvement in overall functioning by week 6 (Axsome Therapeutics, 2020b). AXS-05 demonstrated encouraging results also in TRD by week 1, with effects on depression, anxiety, and psychosocial functioning lasting up to 12 months (ClinicalTrials.gov Identifier NCT04634669) (Axsome Therapeutics, 2022). The drug consistently reduced the risk of depressive symptoms relapse (ClinicalTrials.gov Identifier NCT04608396) (Axsome Therapeutics, 2021). The primary antidepressant action of this drug was associated with an enhancement in cognitive function (Axsome Therapeutics, 2020c), a durable improvement in psychosocial functioning (Axsome Therapeutics, 2022), as well as a rapid and progressive relief of anxiety symptoms associated with TRD (Axsome Therapeutics, 2020c, 2022); however, one RCT in a well powered sample did not confirm AXS-05 benefits in TRD in a 12-week study (ClinicalTrials.gov Identifier NCT02741791) (Axsome Therapeutics, 2020c). An additional RCT is currently ongoing and aims to evaluate AXS-05 for MDD not responding to standard antidepressants and MDD with suicidal ideation (ClinicalTrials.gov Identifier NCT04971291) (Clinicaltrials.gov, 2021c). To summarize, AXS-05 has good evidence of efficacy on overall depressive and anxiety symptoms but targets well also suicidal ideation and cognitive dysfunction, two symptoms that often do not respond well to standard antidepressants, with resulting benefits on functioning in relevant areas of life.

### Compounds acting on the serotonergic system

#### Psilocybin

Psilocybin (4-phosphoryloxy-N,N-dimethyltryptamine) is a tryptamine alkaloid hallucinogen naturally present in the psilocybe genus of mushrooms (Passie *et al.*, 2002). Its antidepressant effect is putatively mediated by the serotonergic system, specifically by serotonin 2_A_ receptors (5HT_2A_Rs) agonism that leads to a major glutamate release by pyramidal neurons that express these receptors in the PFC. This activation results in a modulation of brain circuit connections (Carhart-Harris *et al.*, 2014); however, the mechanisms behind the persistence over time of these neuroplasticity phenomena remain unknown. Imbalances of serotonin in the brain underpin and modulate depression, as well as emotional and physical symptoms of anxiety, cognition, learning, memory, reward processing, and sleep (Pourhamzeh *et al.*, 2022).

Psilocybin has been studied for the treatment of MDD, but also for depression associated with general medical comorbidities. In addition to its potential therapeutic role in mood disorders, psilocybin showed preliminary benefits in obsessive-compulsive disorder (Moreno *et al.*, 2006) and substance dependence (Veen *et al.*, 2017).

All studies evaluated the effects of psilocybin administered in supportive and controlled environments, in combination with behavioural psychological support provided by trained therapists (psilocybin-assisted psychotherapy, PAP).

##### Major depressive disorder

PAP may represent a successful option for reducing depressive symptoms, with or without anxiety, with rapid effects lasting for about 6 months. This efficacy was confirmed in a recent meta-analysis of four small trials (Goldberg *et al.*, 2020). Psilocybin was recognized as a breakthrough therapy for TRD by the FDA in 2018 and for MDD in 2019.

Positive effects of psilocybin in TRD, especially for the dose of 25 mg/day, are supported by a pilot study (ISRCTN14426797) (Carhart-Harris *et al.*, 2016) and two well powered RCTs (ClinicalTrials.gov Identifier: NCT03775200 and NCT04670081, which is still ongoing) (Clinicaltrials.gov, 2021b; Compass Pathways, 2021). The results showed a rapid reduction of depressive and anxiety symptoms (by week 1 and week 3) and sustained improvement until months 3 and 6 in some participants. Importantly, anhedonia improved together with depressive symptoms (Carhart-Harris *et al.*, 2016). Similarly, pessimism bias (unrealistic pessimism when predicting the occurrence of future life events) is a manifestation deeply connected with severe depression and decreased after administration of psilocybin, as reported in a post-hoc study with a small sample size of patients with TRD (Lyons and Carhart-Harris, 2018). Consistent with these cognitive changes, in a recent study psilocybin showed benefits on cognitive empathy in TRD patients with a subsequent improvement in emotional face recognition; this effect correlated also with reduced anhedonia (Stroud *et al.*, 2018). These findings suggest that psilocybin may enhance cognitive flexibility (the capacity to adapt thoughts and behaviours to multiple environmental demands), as confirmed in another study, with an improvement found to persist for at least 4 weeks posttreatment (ClinicalTrials.gov Identifier NCT03181529) (Doss *et al.*, 2021). Therefore, psilocybin seems to act positively on symptoms poorly targeted by standard antidepressants, but another important question is if it is overall superior to standard antidepressants in terms of depressive symptoms improvement. According to two RCTs, psilocybin is not more beneficial than escitalopram on overall depressive symptoms over a short period of time, but a different profile of action on depressive symptoms was confirmed. In a 6-week study (ClinicalTrials.gov Identifier NCT03429075) (Carhart-Harris *et al.*, 2021), two administrations of psilocybin improved the perceived ability to cry and to feel compassion and intensified emotion and pleasure compared to escitalopram, as well as reduced suicidal ideation more than then escitalopram. In a crossover study (ClinicalTrials.gov Identifier NCT03912974) (Becker *et al.*, 2022), a single dose of psilocybin was administered after a 2-week pretreatment with escitalopram or placebo. Escitalopram reduced psilocybin-induced adverse events [increased blood pressure and heart rate (HR), pupil dilatation, and other acute autonomic events], and negative psychic effects such as anxiety (‘anxious ego-dissolution’); however, escitalopram did not show additional efficacy on positive mood effects of psilocybin versus placebo. Finally, various phase I (ClinicalTrials.gov Identifier: NCT03554174) and phase II RCTs (ClinicalTrials.gov Identifier: NCT03866174, NCT03715127, NCT04620759, NCT04630964, NCT03380442) are currently ongoing. These trials aim to confirm the efficacy of psilocybin on depressive symptoms and some of them will also investigate neuroplasticity changes and brain mechanisms underpinning these changes (Clinicaltrials.gov, 2018, 2019a, 2019b, 2021d, 2021e, 2021f).

##### Major depressive disorder associated with general medical comorbidities or life-threatening illnesses

There is strong evidence supporting the efficacy of psilocybin on depressive and anxiety symptoms associated with life-threatening illnesses, such as neoplastic diseases or AIDS.

Clinical trials focused on psilocybin use in patients with advanced-stage cancers, located in the breast, colon, ovary, peritoneum, or salivary gland. Lymphoma, leukemia, and myeloma were also included (Doblin *et al.*, 2019). Robust efficacy was confirmed in two systematic reviews as well (Reiche *et al.*, 2018; Ross, 2018). The antidepressant and anxiolytic effects of psilocybin were immediate and persisted in a large percentage of patients for at least 6 months after a single active treatment [ClinicalTrials.gov Identifier: NCT00465595 (Griffiths *et al.*, 2016) and ClinicalTrials.gov Identifier: NCT00957359 (Ross *et al.*, 2016)] or even up to 4.5 years following a single dose, as demonstrated by a long-term follow-up study (Agin-Liebes *et al.*, 2020). Such enduring improvements in anxiety and depression led to a consequent reduction in demoralization, hopelessness, and death anxiety (Ross *et al.*, 2016; Agin-Liebes *et al.*, 2020) and an improvement in optimism, death acceptance, and quality of life (Griffiths *et al.*, 2016); however, these positive findings followed the administration of a high dose of psilocybin (22 or 30 mg/70 kg) and did not occur with a low dose (1 or 3 mg/70 kg) (Griffiths *et al.*, 2016), in line with the results of a pilot study (ClinicalTrials.gov Identifier: NCT00302744). Only trait anxiety decreased at months 1 and 3, whereas a significant decrease in measures of depression was demonstrated only at month 6 (Grob *et al.*, 2011). Of note, there are reports of a major reduction in negative emotionality and increased creativity when assuming psilocybin micro-dosing rather than a single active treatment (Anderson *et al.*, 2019). For micro-dosing, the FDA refers to a dose of the drug that is 1% of the pharmacologically active dose, up to a maximum of 100 µg (EMA 2003); however, no advantage of micro-dosing over placebo was found in patients with depressive/anxious disorders not related to neoplastic diseases (Marschall *et al.*, 2022), while one RCT reported benefits in patients with life-threatening cancer. In this study, a single dose of psilocybin was associated with acute and sustained reductions in suicidal ideation as early as 8 h and sustained for 6.5 months posttreatment. Loss of meaning (LoM), a component of demoralization, had similar reduction as early as 2 weeks after treatment and was sustained at 4.5 years of follow-up. A positive relationship was found between reductions in LoM and suicidal ideation, and between suicidal ideation and hopelessness and demoralization (Ross *et al.*, 2021). These encouraging results prompted another recent pilot study (ClinicalTrials.gov Identifier: NCT02950467), that showed robust reductions in self-reported demoralization, grief, and psychological trauma in AIDS survivors. Over 7 weeks, patients were treated with both individual and group psilocybin sessions (8 and 12–15 h respectively). The self-reported demoralization, the primary clinical outcome, linearly decreased from baseline to the last follow-up assessment point at month 3 (Anderson *et al.*, 2020).

#### Pimavanserin

Pimavanserin is an atypical antipsychotic that does not directly target dopamine receptors (Rissardo *et al.*, 2022). Indeed, it predominantly acts on the serotonergic neurotransmission, through an inverse agonism and antagonism on the 5-HT_2A_Rs, and the 5-HT_2C_Rs at higher doses (Vanover *et al.*, 2006). It is approved by the FDA to treat hallucinations and delusions associated with psychosis in Parkinson’s disease with a recommended dose range between 34 and 40 mg once daily.

##### Major depressive disorder

In light of the serotonergic profile, pimavanserin effectiveness was also tested in mood disorders, particularly in MDD. Short- to medium-term benefits (weeks 1–5) on depressive symptoms were demonstrated by the first stage of one RCT performed in a well powered sample (Clinical trial identification number NCT03018340); however, the second stage did not confirm these positive results, probably due to the smaller sample size (Fava *et al.*, 2019). In this study, pimavanserin was administered as adjunctive treatment to standard antidepressants (SSRI or SNRI) and produced benefits on depressive symptoms that did not respond to previous antidepressant treatments. Moreover, significant improvement was observed for several secondary endpoints including impulsivity, irritability, anxiety, insomnia and daytime sleepiness, sexual and psychosocial functioning, and quality of life. In this regard, results from a secondary analysis have shown a reduction of anxiety and somatic symptoms in a sub-group of patients with severe and anxious MDD at baseline (Papakostas *et al.*, 2020). Of note, in another secondary analysis, pimavanserin reduced both night sleep impairments and excessive day sleepiness and improved global functioning. Therefore, the efficacy of pimavanserin on psychosocial functioning may at least partly be mediated by the reduction of excessive daytime sleepiness (Jha *et al.*, 2020). Interestingly, according to another post-hoc analysis, sexual function improved after treatment with pimavanserin. Women reported increased sexual desire, sexual arousal, orgasmic propensity, and general sexual satisfaction; similar results were not observed in male participants, probably due to the small number of men in the sample (Freeman *et al.*, 2020). Noteworthy, the results of a secondary analysis demonstrated that pimavanserin determined a greater reduction in suicidal ideation by week 3 compared to placebo, despite the exclusion of patients with a history of previous suicide attempts and the small number of participants who reported suicidal ideation during the study (Shelton *et al.*, 2020). Despite these promising results, three subsequent trials (Clinical trial identification number NCT03968159, NCT03999918, and NCT04000009) did not find any antidepressant effect, in the short or in the long term, and the sponsor decided not to pursue the development of pimavanserin for MDD; however, among secondary outcomes, an improvement in subjective quality of sleep was reported (Acadia, 2020).

##### Major depressive disorder associated with general medical comorbidities

Notwithstanding pimavanserin was withdrawn from development for MDD, there is currently one trial supporting potential benefits in reducing depressive occurrences in Parkinson’s disease, in the short-medium term (week 8), both in monotherapy and as adjunctive therapy to standard antidepressants (SSRI or SNRI) (ClinicalTrials.gov Identifier NCT03482882). This study also reported amelioration in secondary clinical outcomes such as global disease severity, sleep quality, overall quality of life, and motor function, though the sample size was small, and the psychometric instrument used in the study (Hamilton Depression Rating Scale or HDRS) was not developed for use in Parkinson’s disease (DeKarske *et al.*, 2020).

### Compounds acting as neuroactive steroids

#### Zuranolone

Zuranolone, an allopregnanolone analogous, is a neurosteroid that displays most of its therapeutic effects acting as a PAM of the synaptic and extra-synaptic GABA_A_Rs. It increases tonic and phasic GABA_A_ currents (Botella *et al.*, 2017). The facilitation of GABA circuits in the central nervous system (CNS) is a common mechanism of action shared also by endogenous neurosteroids (Abramian *et al.*, 2014). Zuranolone is under investigation as a monotherapy for MDD (including PPD) and bipolar depression, using a treatment paradigm of 2 weeks. Its long half-life allows once-daily oral dosing, facilitating its administration and enhancing the therapeutic adherence (Althaus *et al.*, 2020).

##### Major depressive disorder

Zuranolone shows antidepressant effects, especially in patients with depression and anxiety features, as demonstrated by three RCTs in well powered samples (ClinicalTrials.gov Identifier NCT03000530, NCT04442490, NCT04476030). Both 30 and 50 mg showed a rapid onset of action at day 3 and major benefits at day 15 in monotherapy (Gunduz-Bruce *et al.*, 2019; Sage Therapeutics and Biogen, 2021b). When it was co-initiated with an SSRI or SNRI, benefits were reported during a 2-week treatment period, but not after, suggesting a nonsustained efficacy over time (Sage Therapeutics and Biogen, 2022). Although in one study the stabilization of symptoms persisted until day 42–45 (Sage Therapeutics and Biogen, 2021b), in another RCT in a larger sample (ClinicalTrials.gov Identifier NCT03672175), zuranolone 30 mg demonstrated benefits only in the very short term, from day 3 to day 12, and the dose of 20 mg was totally ineffective. Post-hoc analysis indicated that approximately 9% of the patients treated with 30 mg were nonadherent to the treatment. When the data were reanalyzed after excluding these patients, the dose of 30 mg improved depressive symptoms at day 15 (Sage Therapeutics, 2019). Zuranolone demonstrated a dose-dependently effect also in an open-label study, where the dose of 50 mg exhibited a stronger and more prolonged effect than the 30 mg dose in reducing depressive core symptoms, regardless of the co-therapy with an SSRI or SNRI (ClinicalTrials.gov Identifier NCT03864614). The majority of patients did not require more than two 2-week treatment cycles to reach remission (Sage Therapeutics and Biogen, 2021a). Zuranolone 30 mg gave initial evidence of effectiveness in cases with residual insomnia, with improvement in objective measures of quality of sleep, for example, total sleep time, latency to persistent sleep, the median number of awakenings, and mean duration of awakenings (ClinicalTrials.gov Identifier NCT03771664 and NCT04007367); however, both studies were terminated to advance the program with the 50 mg dose (Sage Therapeutics, 2022).

##### Postpartum depression

Zuranolone, both 30 and 50 mg doses, showed encouraging results as a treatment for PPD in two RCTs with well powered samples of outpatient women with sever mood impairment (ClinicalTrials.gov Identifier NCT02978326 and NCT04442503). The first trial included a sample of women who were up to the sixth month postpartum and had depression with onset between the last trimester and 4 weeks after delivery. Rapid (as soon as day 3) and persistent antidepressant effects (day 45) were demonstrated after 2 weeks of treatment with zuranolone 30 mg, as well as rapid improvement in anxiety, global functioning, and self-reported maternal function, despite a high placebo response (Deligiannidis *et al.*, 2021). In the second trial, zuranolone 50 mg confirmed benefits on depression and anxiety, assessed at the same time points (Clinicaltrials.gov, 2020d); however, the possible persistence of the effect beyond 45 days and the long-term safety and efficacy are currently unknown.

##### Bipolar depression

Zuranolone 30 mg may provide rapid (by day 3) and prolonged (as far as day 42) relief of depressive symptoms in patients with bipolar depression type I or II, with a current major depressive episode, as suggested by an exploratory open-label study (ClinicalTrials.gov Identifier NCT03692910). In this study, the 2 weeks treatment paradigm was used, and two patients reported mild hypomania in the follow-up period, but no cases of mania, increased suicidal ideation, or suicidal behaviour were observed (Gunduz-Bruce *et al.*, 2020). There is still no evidence of potential benefits for anxiety or insomnia associated with bipolar depression.

#### Prax-114

Prax-114 is a novel extra-synaptic GABA_A_Rs PAM, currently in phase II of development for the treatment of MDD (Hecking *et al.*, 2021). In-vitro models it was demonstrated that PRAX-114 potentiates GABA-induced currents at both extra-synaptic and synaptic GABA_A_Rs, but with 10.5-fold greater potentiation of the former compared to the latter Rs. This pharmacodynamic property was suggested to potentially reduce the risk of sedative side effects, providing a wider therapeutic window versus other GABA_A_Rs PAMs, while keeping the antidepressant and anxiolytic benefits (Hughes *et al.*, 2021).

Despite this promising pharmacodynamic mechanism, to our best knowledge PRAX-114 did not show any antidepressant effect in MDD when tested as monotherapy in a well powered RCT (ClinicalTrials.gov Identifier NCT04832425) (Praxis, 2022). Another well powered RCT (ClinicalTrials.gov Identifier NCT04969510) will evaluate the efficacy of PRAX-114 10–20–40–60 mg compared to placebo as adjunctive therapy to the current antidepressant treatment but no results are available (Clinicaltrials.gov, 2021a). Efficacy of PRAX-114, apart from mood disorders, is investigated also in posttraumatic stress disorder and essential tremor.

##### Menopausal and mood symptoms

There is preliminary evidence of efficacy of PRAX-114 in menopausal and mood symptoms. An open-label study used a single daily 60 mg dose in a small sample of outpatient women with menopausal distress and PMD. Prax-114 gradually improved mood symptoms and decreased moderate-to-severe vasomotor symptoms (e.g. hot flashes), in a 2-week treatment paradigm. Participants reported enhancement in energy and volition, sexual interest, sleep, anxiety, and irritability; however, symptoms worsened when treatment was discontinued, suggesting the need for a longer therapy (Praxis, 2021); however, these results were not published in a peer-reviewed journal, and apparently, the study was not preregistered.

#### Ph-10

Ph-10 (pregn-4-en-20-yn-3-one) is a neuroactive steroid that belongs to the group of ‘pherines’. Its intranasal administration engages local nasal chemosensory receptors that connect the olfactory system with limbic circuits through oligosynaptic neural connections (Monti *et al.*, 2019). Extensive evidence demonstrated the fundamental role of the human olfactory system in social interactions, vegetative functions, emotions, and mood (Pause *et al.*, 2001; Krusemark *et al.*, 2013). Ph-10 demonstrated a good safety and tolerability profile versus placebo in a dose escalation study (from 0.8 to 6.4 µg), in a small sample of healthy volunteers (Monti *et al*., 2019). To the best of our knowledge, only one RCT testing this drug in MDD was published. The study included patients with moderate MDD and showed that self-administrated intranasal PH-10 at high dose (6.4 µg) had antidepressant effects by week 1, which were sustained in the short-to-medium-term (up to the study endpoint at week 8). A small dose-dependent effect favoured the high dose over the low dose (3.2 µg). Amelioration of depressive symptoms was not strongly supported by a significant improvement in quality and life satisfaction. These results cannot be generalized to patients with suicidal ideation, TRD, and bipolar depression, as these groups were excluded from the trial (Monti *et al.*, 2019). Possible benefits of anhedonia, anxious or irritability features, sleep disturbances, or other specific depressive domains were not investigated.

### Compound acting on the orexinergic system

#### Seltorexant (JNJ-42847922/MIN-202)

Seltorexant is a highly selective antagonist of the human orexin-2 receptor (OX-2R). There are two different orexin types: orexin A (OX-A) and orexin B (OX-B). Both are excitatory neuropeptide hormones, also known as hypocretins, that derive from a common pre-pro-orexin precursor produced in the posterior lateral hypothalamus. Their target is represented by orexin-1 receptor (OX-1R) and OX-2R, which are differently expressed through the CNS. OX-A displays a similar affinity for both receptors, and OX-B shows a greater affinity for OX-2R (Nollet and Leman, 2013).

The orexin system is increasingly being recognized as a key factor involved in hyperarousal and wake/sleeping transition and disturbances, with important implications in stressful or threatening situations. Orexins are involved in multiple functions often compromised during depressive episodes, such as the regulation of circadian rhythms, cognitive (including attention, learning, and memory) and sexual functioning, positive emotions and social interactions, hedonic capabilities and reward process, visceral functions, sensory systems, and pain (Nollet and Leman, 2013), as well as in anxiety and panic states (Johnson *et al.*, 2012). Seltorexant is in phase III of development for MDD and has shown efficacy in normalizing sleep (Bonaventure *et al.*, 2015). In an exploratory clinical study (Clinical trial identification number NCT02067299), the drug demonstrated a dose-dependent normalization of sleep after a single dose of 10–20 or 40 mg in MDD patients treated with SSRI or SNRI with residual insomnia (Brooks *et al.*, 2019); the dose-dependent effect is in line with previous studies of OX2R antagonists in heathy subjects (Ark *et al.*, 2018). The aforementioned study was not designed to test the possible antidepressant effect of seltorexant; however, it was reported that the 40 mg dose as monotherapy improved mood compared with other dosages. In a relatively small sample of patients with mild to moderate depression (Clinical trial identification number NCT02476058), seltorexant showed antidepressant effects as early as day 11 of treatment with 20 mg, with an improvement in self-reported sleep quality. The antidepressant efficacy was sustained with continued treatment for up to 28 days (Recourt *et al.*, 2019). Higher benefits on core symptoms of depression and anxiety were demonstrated by seltorexant as adjunctive therapy to ongoing antidepressants in patients with MDD (Clinical trial identification number and NCT03227224). During these 6-week-period studies, the 20 mg dose showed higher antidepressant efficacy than the 10 and 40 mg doses, and it was even more effective in patients having sleep disturbances. The 40 mg dose improved sleep impairments, but it showed lower efficacy on depressive symptoms (Savitz *et al.*, 2021). This result is in contrast with the findings previously mentioned, therefore it remains unclear which dose is more effective in case of residual insomnia in MDD.

Despite the positive results presented, one long follow-up RCT in a well powered sample did not find any benefit of seltorexant on depression and anxiety, with or without insomnia, when 20 and 40 mg were tested as adjunctive treatment to the ongoing antidepressant versus quetiapine extended-release 150–300 mg (ClinicalTrials.gov Identifier NCT03321526) (Clinicaltrials.gov, 2017).

Other phase II and phase III studies in adolescent, adult, and elderly patients with residual insomnia in MDD are ongoing (Clinical trial identification number NCT04532749, NCT04533529 NCT04451187, NCT04513912, NCT04951609) (Clinicaltrials.gov, 2020a, 2020b, 2020c, 2020e, 2021d). These studies will test seltorexant as adjunctive therapy to the ongoing antidepressant. To our current knowledge, there is no evidence of efficacy for seltorexant in TRD.

### Adverse events, safety, and tolerability

Considering the impact of the safety profile on treatment adherence, in this paragraph we provide an overview of the most frequent adverse events associated with the drugs of interest, to guide the personalization of treatment choice also based on the tolerability profile, and to properly inform patients.

**Fig. 2:**
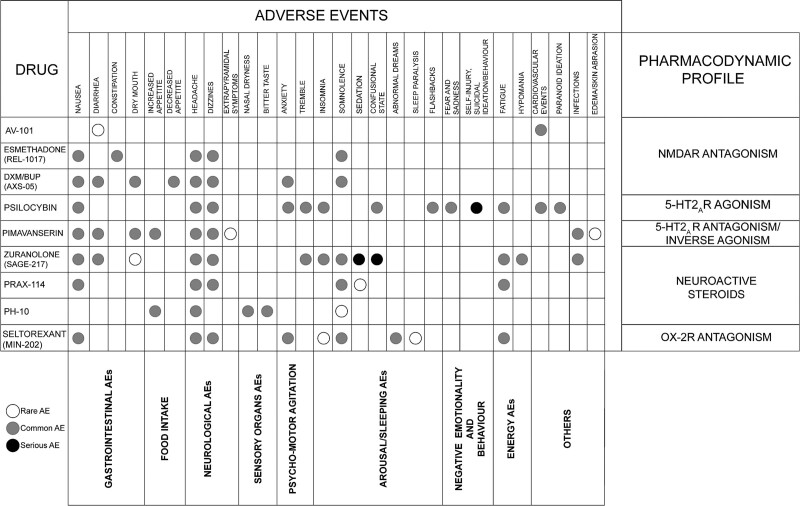
Description of adverse events of novel antidepressant drugs. Common adverse events were defined as those having a frequency ≥5%, while rare adverse events as those with a frequency <5%. Serious adverse events are defined as any adverse event causing clinically significant disability and incapacity or immediately life-threatening. BUP, bupropion; DXM, dextromethorphan; NMDAR, n-methyl-d-aspartate receptor; OX-2R, orexin type 2 receptor; R, receptor.

Overall, the discussed medications were safe and well tolerated, with a few serious adverse events. Details on the adverse events profile of each drug of interest are reported in Fig. 2; however, there is still limited evidence, particularly in the long term, and longitudinal studies are needed.

The neurological safety profile appears to be the most affected by new rapid agents. Headache was one of the most frequently reported adverse events for all compounds (apart from AV-101), with a frequency exceeding 10%; the risk seems to be more elevated in subjects exposed to psilocybin (67%) (Carhart-Harris *et al.*, 2021) and seltorexant (40%) (Brooks *et al.*, 2019). Dizziness was reported by 25% of patients treated with seltorexant (Brooks *et al.*, 2019), despite being a common adverse event of other medications, with a frequency higher than 5%. Extrapyramidal symptoms (EPS), that is, akathisia, bradykinesia, and rigidity, were observed for pimavanserin, and ranged from 1.3% to 3.4%, without compromising motor function even in patients with Parkinson’s disease (DeKarske *et al.*, 2020). Tremor is another adverse event that might represent an EPS or be associated with psychomotor agitation. In patients receiving psilocybin, it was mainly coupled with fear, sadness, and a physical experience of feeling the body shake (Doss *et al.*, 2021). In patients treated with zuranolone, it was often associated with insomnia in 7% of patients and, similarly to the other adverse events of zuranolone, occurred predominantly during the 2-week treatment paradigm and then was no longer reported (Sage Therapeutics and Biogen, 2021a).

Pimavanserin and AV-101 appeared to be the safest with respect to psychomotor agitation, arousal and sleeping disturbances. On the other hand, 17–23.2% of patients with MDD and life-threatening illnesses treated with psilocybin, especially at higher doses, developed a mild activation syndrome at the beginning of treatment, mainly characterized by anxiety (Griffiths *et al.*, 2016; Ross *et al.*, 2016); however, in one trial anxiety affected 100% of patients (Carhart-Harris *et al.*, 2016). Anxiety was generally acute and transient, but it also appeared several days after treatment (Anderson *et al.*, 2020). It was mainly attributable to the psychological discomfort during psilocybin sessions, and it improved with reassurance from the clinical team.

Rates of insomnia were found to be over 10% for psilocybin and were sometimes accompanied by posttraumatic stress flashbacks (Anderson *et al.*, 2020). In patients treated with seltorexant, few cases of unexpected insomnia (2.1%), sleep paralysis (1.4%), abnormal dreams (13%), and nightmares (1.4%) led to treatment discontinuation (Recourt *et al.*, 2019; Savitz *et al.*, 2021). In another trial of seltorexant, up to 13% of patients experienced abnormal dreams (Clinicaltrials.gov, 2017). No hypnagogic nor hypnopompic hallucinations were observed and there was no clear evidence of dose-related effect on these adverse events. Seltorexant safety profile seems similar to the first-in-class orexin receptor antagonist, namely suvorexant, already approved by FDA for the treatment of insomnia (Kuriyama and Tabata, 2017); however, unlike seltorexant, suvorexant has a longer half-life (about 12 h), with more severe adverse events, higher rates of sleeping disturbances (Sutton, 2015), as well as a functioning impairment, caused by symptoms similar to cataplexy that may occur the day after treatment (Recourt *et al.*, 2019).

Although somnolence was common for different compounds, there are substantial differences. For seltorexant, somnolence appeared to be dose-related within the dose range of 10–40 mg/day, and was reported by 25% of patients (Brooks *et al.*, 2019); however, somnolence was most frequent in patients treated with the GABA_A_Rs modulators PRAX-114 and zuranolone, with maximum rates of 37.5% and 26.5%, respectively (Clinicaltrials.gov, 2020d; Praxis, 2021). Despite resolving within a few hours, sedation may represent a serious adverse event for both compounds, especially when associated with confusion (Deligiannidis *et al.*, 2021; Praxis, 2021). Transient confusion induced by psilocybin, albeit frequent (75%), can probably be considered a psychological effect that subsided after a few hours (60–180 min) (Carhart-Harris *et al.*, 2016). These sleeping and arousal adverse events must be differentiated from fatigue, intended as the lack of energy and motivation to perform daily activities. Although it often associates with the daytime somnolence and sedation induced by GABAergic and orexinergic compounds, fatigue achieves higher rates during psilocybin trials (7–10%), suggesting more complicated mechanisms that extend beyond the alteration of the hypnic pattern (Carhart-Harris *et al.*, 2021; Compass Pathways, 2021).

Regarding cardiovascular adverse events, psilocybin, and AV-101 caused minimal reductions in SBP/DBP and basal HR that can be related to a mild sympathomimetic effect (Grob *et al.*, 2011; Griffiths *et al.*, 2016; Ross *et al.*, 2016; Doss *et al.*, 2021). For AV-101 these events were not generalizable (Murphy *et al.*, 2021), whereas patients receiving psilocybin reported cardiovascular adverse events in 76% of cases according to one RCT (Ross *et al.*, 2016). No effects on QT interval prolongation emerged for any of the considered drugs.

Gastrointestinal adverse events were common for all the discussed agents. Psilocybin was associated with the highest rates of nausea (33%) (Carhart-Harris *et al.*, 2016) and REL-1017 with the highest rates of constipation (14%), showing a dose-response relationship probably linked to the weak stimulation of opioid receptors (Fava *et al.*, 2022). Dry mouth was a frequent adverse event experienced by 10.3% of patients treated with AXS-05 (Tabuteau *et al.*, 2022) and by 9.6% of subjects with pimavanserin (Fava *et al.*, 2019). Conversely, PH-10 and AV-101 were less likely to cause gastrointestinal adverse events: PH-10 showed a low trend to increase appetite that did not result in weight gain (Monti *et al.*, 2019) and only one participant experienced transient diarrhoea with AV-101 1440 mg (Murphy *et al.*, 2021). In general, more than 5% of patients treated with the other compounds experienced gastrointestinal adverse events, without resulting in hospitalization or treatment discontinuation. No gastrointestinal bleeding was reported.

Despite the limited data concerning PH-10, local nose irritation due to the intranasal administration, and bitter taste were reported during the treatment period (Monti *et al.*, 2019). No other adverse events related to sensory organs were reported for the other compounds.

Considering all the discussed medications, there was no evidence of abuse or dependency potential, increased suicidal ideation, and/or suicidal behaviour compared with baseline. Only in one trial of psilocybin, 12 patients reported suicidal ideation or suicidal behaviour and intentional self-injury, which are however quite common in patients with TRD (Compass Pathways, 2021). During treatment with zuranolone, one case of suicide attempt and one of bile duct stones were reported; however, these patients had a previous history of suicide attempt and of bile duct repair, respectively (Sage Therapeutics, 2019).

Participants exposed to the discussed NMDAR antagonists experienced no worsening in depression, dissociative or psychotomimetic effects. In reference to this, it has been hypothesized that the unusually rapid rate of NMDAR unblocking may explain the low association of these drugs with psychotomimetic adverse events (Parsons *et al.*, 1995). Only after psilocybin administration, a few cases of paranoid ideation and transient thought disorders were described, but they were generally mild and transient (Ross *et al.*, 2016; Anderson *et al.*, 2020); only in one trial 75% of participants underwent through a transient thought disorder (Carhart-Harris *et al.*, 2016). No hallucinogen-persisting perception disorder or visual perceptual changes were observed, probably because of the safe environment wherein psilocybin sessions were tested. Accurately informing patients about possible adverse events contributed to avoiding psychotic reactions (Studerus *et al.*, 2012).

Another noteworthy safety consideration is the low tendency of the drugs of interest to produce clinically significant changes in vital signs or consciousness, sexual dysfunction, cognitive impairment, euphoria, or manic episodes (only two cases of hypomania occurred during the follow-up period in the trial testing zuranolone in bipolar depression) (Gunduz-Bruce *et al.*, 2020), withdrawal symptoms and weight gain or metabolic changes. Despite the time of observation being fairly limited to make firm conclusions, novel antidepressant drugs may represent metabolically and sexually friendly antidepressants. The relevance of the latter issue, among the general safety profile, acquires even more importance considering the limitations in the use of conventional antidepressants because of weight gain (Serretti and Mandelli, 2010), sexual impairments (Serretti and Chiesa, 2009) and (hypo)manic switches when used in bipolar depression (Bauer, 2022).

## Discussion

**Fig. 3:**
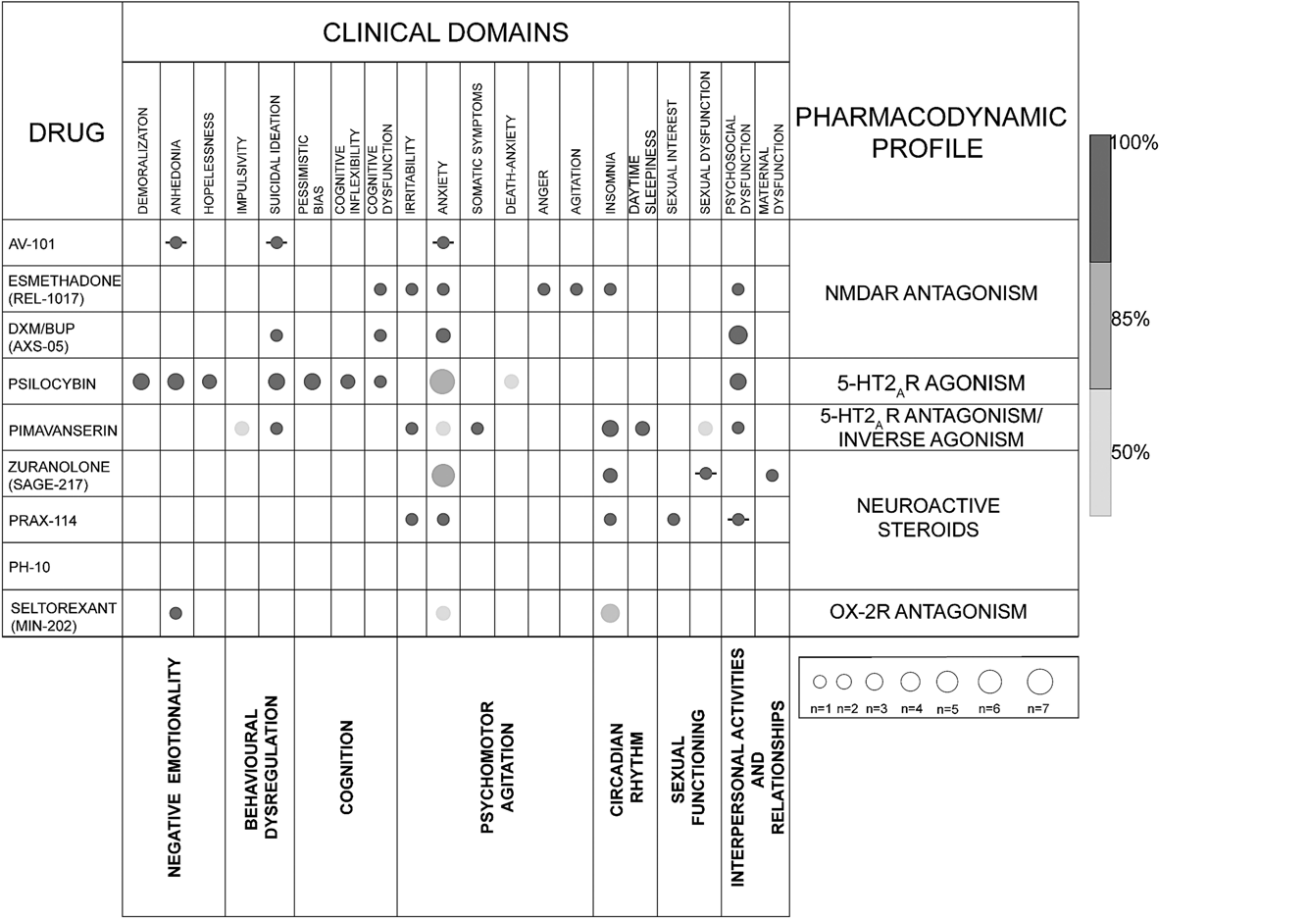
Clinical features targeted by novel antidepressant drugs based on the availability of studies investigating a specific symptom domain even as a secondary outcome. An empty box indicates no studies exploring a specific symptom, while a larger diameter of dots indicates a progressively higher number of studies. Darker shades of grey indicate a progressively higher percentage of the number of studies reporting a positive result referred to the total number of studies investigating the specific symptom. Barred dot indicates studies with negative results in the explored symptom. BUP, bupropion; DXM, dextromethorphan; NMDAR, n-methyl-d-aspartate receptor; OX-2R, orexin type 2 receptor; R, receptor.

Rapid acting antidepressants represent promising alternatives to standard medications for mood disorders, with various levels of evidence and in different phases of development, with overall encouraging findings about their efficacy and tolerability. This review provides an overview of the clinical trials that investigated new rapid acting antidepressants, with the aim to provide clinically useful information to tailor the prescription of these new compounds, based on the individual clinical profile. The specific clinical features targeted by each novel antidepressant drug were identified according to the current evidence and are reported in Fig. 3. There is not sufficient evidence to demonstrate if AV-101 and PH-10 positively target any specific depressive symptoms. Among new drugs acting on the glutamatergic system, we discussed AV-101, AXS-05, and REL-1017. Despite these three compounds primarily targeting the glutamatergic signal, AV-101 and AXS-05 appear to exhibit a major involvement in inflammatory pathways underpinning mood disorders, while the antidepressant action of REL-1017 seems to be primarily related to neuroplasticity phenomena, since the persistence of the therapeutic effect after treatment discontinuation.

Av-101 principally acts through the kynurenine pathway, which plays an important role in various biological functions behind depression. Additionally, it also modulates brain-derived neurotrophic factor (BDNF) levels (Réus *et al.*, 2015). Response to this drug appears to be compromised by comorbid anxiety disorders (Park *et al.*, 2020) unless used as adjunctive treatment to standard antidepressants or with an appropriate dose increment. Higher doses (1440 mg/day) are indeed potentially more effective than lower doses (720 mg/day) in a preclinical study (Murphy *et al.*, 2021). To further increase bioavailability, despite not overcoming the limitations reported in animal studies, the combination of AV-101 with probenecid (a uricosuric agent) has recently been investigated in rodents, with safe results (Murphy *et al.*, 2021). Despite the drug was not associated with an improvement in suicidal ideation and/or suicidal behaviour, it was hypothesized to be a promising option for suicidality treatment in emergency settings, because of its ketamine-like glutamatergic mechanism of action, and the lack of dissociative adverse events (Zanos *et al.*, 2015). Additionally, its antisuicidality effect may be not limited to the acute phase. Research in cases of suicide attempts shows that pro-inflammatory cytokines, frequently increased in these patients, lead to a persistent increase in quinolinic acid, which binds to NMDAR, and to a decrease in kynurenic acid (Bay-Richter *et al.*, 2015). Consequently, AV-101 may rebalance quinolinic and kynurenic acids equilibrium, displaying a protective action on suicide risk also in the medium-long term.

Rel-1017, another NMDAR antagonist, demonstrated rapid efficacy in MDD, with the persistence of antidepressant activity up to 1 week after treatment discontinuation (Fava *et al.*, 2022). Additionally, changes in anhedonia, motivation, and reward have accompanied antidepressant-like activity in rodent models, after a single dose (Fogaça *et al.*, 2019). Rel-1017 may represent therefore a valid therapeutic option not only in MDD but also in TRD. Importantly, the potential of abuse should be better explored since the low affinity to the opioid receptors does not automatically exclude this issue (Nemeroff, 2022).

AXS-05, presumably due to the multimodal pharmacological activity, was beneficial in rapidly reducing depressive symptoms, even when associated with anxiety features and when unresponsive to other antidepressants, both in the short (by week 1) and long term (up to 12 months). Noteworthy AXS-05, albeit in a limited way, reduced cognitive dysfunctions and produced a tangible improvement in work/family functioning, ameliorating life quality (Axsome Therapeutics, 2020c, 2022). Among the pharmacodynamic mechanisms beyond AXS-05 efficacy in MDD, there is also a potential action towards α-amino-3-hydroxy-5-methyl-4-isoxazolepropionic acid receptors (Nguyen and Matsumoto, 2015), shared also by ketamine (Maeng *et al.*, 2008) and esketamine, approved by the FDA in 2019 for adults with TRD (Stachowicz and Sowa-Kućma, 2022). These two compounds demonstrated clear evidence of efficacy in reducing the severity of suicidal ideation with active intent (Siegel *et al.*, 2021), as well as AXS-05 was reported to contrast suicidal ideation since the first week of therapy (Axsome Therapeutics, 2020b). Dextromethorphan itself has also evidence of preliminary efficacy in bipolar depression, both as monotherapy (Majeed *et al.*, 2021) and as add-on therapy with memantine (Lee *et al.*, 2020). Consequently, although AXS-05 clinical profile appears to be useful only in MDD and has not yet been investigated in bipolar depression, it owns the potential of being pursued for the treatment of bipolar depression, especially considering the efficacy in the management of cognitive dysfunction and suicidal ideation; however, it remains to be clarified whether AXS-05 efficacy may be extended to other domains typical of depressive experiences, such as anhedonia, demotivation, and alterations in reward processing. According to several studies reporting a correlation between the reduction of anhedonia and suicidal ideation (Ballard *et al.*, 2017), we can hypothesize that AXS-05 may improve anhedonia.

Other drugs discussed in this review, namely psilocybin and pimavanserin, act mostly by modulating the serotoninergic system. Psilocybin in conjunction with psychotherapy demonstrated efficacy within 8 h of taking a single dose in the alleviation of demoralization and hopelessness (Ross *et al.*, 2021). Initial self-perceived effects occur in approximately 30–60 min upon oral administration (Griffiths *et al.*, 2006), and according to most trials, positive effects on anxiety and depressive experiences persist for up to 6 months after a few psilocybin sessions. Such long-lasting positive changes may be related to psilocybin-induced synaptic remodelling. Consistent with psilocybin-induced neuroplasticity, in some trials patients found relief in reconnecting to their emotionality (Anderson *et al.*, 2019; Carhart-Harris *et al.*, 2021; Ross *et al.*, 2021) and improved cognitive flexibility (Griffiths *et al.*, 2016; Doss *et al.*, 2021) that may facilitate, potentially after a single dose, the recognition of feelings (Stroud *et al.*, 2018), thus improving cognitive empathy. Additionally, one RCT in healthy individuals outlined the enhancement in emotional empathy (Pokorny *et al.*, 2017). As multiple deficits in empathic abilities are extremely common in mood disorders and lead to social impairments (Cusi *et al.*, 2011), these pro-empathic properties may be particularly relevant to enhance social skills in patients with MDD, TRD, and depression associated with neoplastic illnesses. In the latter group of patients, anxious and depressive symptoms are often accompanied by cognitive inflexibility with hopelessness, negative cognitive bias, and suicidal ideation. In these cases, as well as in TRD, the rapid and sustained reduction in suicidal ideation of psilocybin is particularly relevant (Carhart-Harris *et al.*, 2021; Doss *et al.*, 2021; Ross *et al.*, 2021). The long-term antisuicidality potential may be linked to the increase of synaptic plasticity and BDNF levels, through modulation of glutamatergic prefrontal-limbic neural circuits. Another pharmacodynamic mechanism potentially behind this antisuicidal effect is the increase of dopamine levels in mesoaccumbens circuits, which may ameliorate reward process impairments (Sakashita *et al.*, 2015). Of note, subjective and mystical experiences are considered to reduce suicidal ideation in the short term (Ross *et al.*, 2021) and to be potentially implied in the risk of abuse (Griffiths *et al.*, 2018); however, the potential of abuse and risk of dependence is fairly reduced by the lack of psilocybin-induced withdrawal and rebound symptoms.

In contrast to psilocybin, pimavanserin exerts an antagonism action mostly towards 5HT_2A_Rs. It showed major efficacy in treating depressive episodes associated with Parkinson’s disease and inducing mood improvement associated with amelioration in sleeping and in overall quality of life (DeKarske *et al.*, 2020). Despite the efficacy of pimavanserin in treating psychic symptoms associated with Parkinson’s disease, its benefits in MDD remain uncertain, due to the negative results of phase III trials (Acadia, 2020). It is however worth mentioning its positive effect on sexual dysfunction. Although it shares monoaminergic activity with other antidepressants, in contrast to them pimavanserin does not exhibit adrenergic, histaminergic, muscarinic, or dopaminergic activity (Vanover *et al.*, 2006), to which sexual dysfunctions are attributed (Serretti and Chiesa, 2009), and the specific 5-HT_2_ antagonism may even improve sexual functioning. Another point to consider is that pimavanserin reduced suicidal ideation more than placebo (Shelton *et al.*, 2020). This antisuicidality potential may be correlated to its efficacy in targeting irritation and insomnia, which in turn are associated with agitation and with depression with mixed features and an increased risk of suicide (McIntyre *et al.*, 2016). To summarize, despite the development of pimavanserin for MDD was discontinued, future studies may clarify if this compound could be a useful option as adjunctive treatment to another antidepressant for MDD with compromised sexual functioning or sexual side effects, anxiety, irritability, agitation, insomnia, or excessive day sleepiness.

Finally, we discussed new compounds acting as neuroactive steroids, which were developed based on the evidence that dysregulation in neurosteroidogenis and GABAergic transmission are involved in anxiety and depressive disorders, including PPD (Luscher *et al.*, 2011).

Zuranolone is a synthetic neurosteroid and targets GABA_A_Rs, similarly to other neurosteroids and benzodiazepines (Goetz *et al.*, 2007); however, zuranolone shows a major affinity to extra-synaptic GABA_A_Rs, responsible for tonic inhibition, rather than synaptic GABA_A_Rs, mediators of phasic inhibition. Consequently, despite sharing with benzodiazepines phasic channel inhibition (clinically resulting in an anxiolytic and sedative action), it differs since it also exerts a tonic inhibition (responsible for the antidepressant action) (Reddy, 2018). This pharmacodynamic profile may hypothetically provide also anxiolytic, hypnotic, and sedative properties. In line with this, the major benefits of zuranolone 50 mg in a 2-week treatment regimen were reported in patients with anxious depression and those with residual insomnia. Efficacy in alleviating sleeping impairments was reported also in a crossover study of healthy adults with sleep disturbances (Bullock *et al.*, 2022); however, improvements in anxiety and insomnia were not confirmed by all trials (Gunduz-Bruce *et al.*, 2019). Despite in a few cases benefits persisted until days 42–45, generally the rapid improvement in MDD symptoms slightly decreased after the end of the 2-week treatment paradigm, thus requiring additional courses of treatment. Zuranolone was also tested in the treatment of PPD, with rapid reduction of depressive symptoms and anxiety, a common feature in PPD (Radoš, 2018), with sustained effects until day 45. The improvement of global and maternal functioning makes zuranolone a valid option for PPD, especially considering that none of the standard antidepressants have a specific indication for this condition. The oral route of administration facilitates the intake compared to its intravenous analogous brexanolone, already approved by FDA in 2019 for PPD. Positive results of zuranolone in bipolar depression open new horizons about the involvement of neurosteroids in this condition. The current evidence suggests that neurosteroids may act as endogenous mood stabilizers and low levels of neuroactive steroids characterize depressive and mixed episodes in bipolar depression. Consistently, preclinical evidence showed that lithium and olanzapine/fluoxetine modify the levels of neurosteroids, and these are all approved therapeutic compounds in bipolar depression (Carta *et al.*, 2012). In summary, zuranolone was effective in reducing depressive symptoms within 15 days of treatment and may represent a viable therapeutic option with a rapid onset of action, especially in agitated depression, depression with anxious features, and sleep impairment. Benefits of zuranolone in TRD and suicidal ideation are still unknown. Of note, co-therapy with benzodiazepines should be avoided, for a consistent risk of increasing sedative effects.

Ph-10 is another neuroactive steroid, that demonstrated a rapid antidepressant potential in unipolar depression; however, only one trial is available, and it is characterized by several limitations that compromised the generalizability of results (Monti *et al.*, 2019). Unlike zuranolone, PH-10 is not a direct GABARs modulator and consequently has a less sedative profile. Early findings demonstrated that ph-94b, another neuroactive compound, had therapeutic potential in patients with social anxiety disorder, because of a rapid decrease in sympathetic tone (Liebowitz *et al.*, 2016). According to the common neurosteroid structure between PH-10 and ph-94b, and to the administration in µg dosages of both agents, similar efficacy of PH-10 in reducing social anxiety could be hypothesized, but it should be tested in future trials. Notably, PH-10 could represent a rapid onset and easy-to-administer (intranasal) option in this group of patients.

Prax-114 is another neurosteroid modulating the GABAergic system, but it currently shows poor evidence of efficacy in treating mood disorders, as it failed in a phase II/III RCT of MDD (Praxis, 2022). This failure underlines the complexity of developing compounds with effective clinical benefits. Despite the failure to demonstrate efficacy in MDD, the drug was tested for treating mood and perimenopausal symptoms in a small preliminary study, suggesting possible improvement in mood, energy, irritability, anxiety, insomnia, and sexual interest, with an overall safety profile (Praxis, 2021). Probably increasing the dosage up to 60 mg, compared with the 40 mg dose used in the MDD trial, contributed to reducing the high rates of insomnia that affect affective changes during menopausal transition, with consequent benefits on mood (Caruso *et al.*, 2019).

Finally, we discussed seltorexant, a novel antidepressant acting on the orexinergic system. This drug demonstrated efficacy as an adjunctive treatment to the ongoing antidepressant in MDD, especially when associated with insomnia. In these cases, apart from showing a major effect on mood, it immediately ameliorated both self-reported and objective parameters of sleep experience within a few hours after administration (Brooks *et al.*, 2019). Improvements in other depressive and anxiety symptoms were not as rapid, but generally started within the first week of treatment, or as early as day 11 according to another study, with the greatest amelioration at weeks 3 and 6 (Savitz *et al.*, 2021). Whether seltorexant displays a dose-dependent effect across doses of 10–20–40 mg remains unclear. In this regard, a curvilinear dose-response relationship was hypothesized as previously noted also for esketamine and nortryptiline, with the maximum antidepressant response at 20 mg and a reduction of the therapeutic effects of seltorexant at the doses of 10 and 40 mg (Savitz *et al.*, 2021). Consistently with the role of orexin pathways in the aetiology of mood, anxiety, and panic symptoms, we suggest that seltorexant might be particularly useful for improving depressive and anxiety symptoms associated with panic distress, as well as for inducing an immediate hypnotic effect. The ideal time of administration should consequently be 1 h before bedtime. These positive effects on sleep may also have benefits in preventing depressive relapses/recurrences, as there is a bidirectional connection between depression and insomnia (Oh *et al.*, 2019). The principal mechanism of alleviating sleeping disturbances consists in reducing sleep latency and increasing sleep duration with very low evidence of altering sleep architecture, thus differing from the currently available hypnotic drugs that significantly alter the rapid eye movement phase (Brooks *et al.*, 2019). Additionally, the involvement of orexin neurons in the modulation of irritability/aggressive behaviours supports the idea of possible effectiveness in these clinical features (Flanigan *et al.*, 2020).

To summarize the clinical specificity profiles targeted by the discussed medications, we considered seven clinical domains: negative emotionality, behavioural dysregulation (impulsive or excessive behaviours that may even put life at risk), cognition, psychomotor agitation, circadian rhythm, sexual dysfunction, and interpersonal activities and relationships (Fig. 3). All compounds, except AV-101 and PH-10, may constitute an appropriate option to reduce psychomotor agitation, and REL-1017 shows the widest profile in terms of the number of targeted symptoms. Psilocybin and zuranolone show the strongest potential to target anxiety. No medication with effectiveness in reducing psychomotor retardation was identified, despite this being one of the main features of severe depressive episodes (Buyukdura *et al.*, 2011). Several drugs (pimavanserin, zuranolone, PRAX-114, and seltorexant) showed efficacy for the regularization of the circadian rhythm, with pimavanserin not only favouring sleeping at night but also reducing excessive daytime sleepiness. Pimavanserin also showed effects on behavioural dysregulation, sexual and global functioning. Psilocybin was found to be the only drug with a strong specificity in reducing negative emotionality and in counteracting pessimism and cognitive inflexibility of depression. It also was effective in reducing behavioural dysregulation. AXS-05 displays a wide profile of efficacy among the domains of behavioural dysregulation, cognition, psychomotor agitation, and interpersonal functioning.

## Limitations and future directions

The results of discussed studies should be interpreted in the context of several limitations. Specifically, the lack of complete data, significant placebo response rates, short-term treatments (Fava *et al.*, 2019), and limited follow-up periods (Clinicaltrials.gov, 2020d; Park *et al.*, 2020; Deligiannidis *et al.*, 2021; Doss *et al.*, 2021) reduced the possibility of finding differences in the experimental arm. Trials with longer follow-ups might be necessary to elucidate long-term antidepressant effects, as well as safety and tolerability in the long term. It should also be noted that some trials were pilot studies (Anderson *et al.*, 2020), with small sample sizes (Ross *et al.*, 2016; Fava *et al.*, 2019; Gunduz-Bruce *et al.*, 2019; Monti *et al.*, 2019; Anderson *et al.*, 2020; DeKarske *et al.*, 2020; Park *et al.*, 2020; Doss *et al.*, 2021; Savitz *et al.*, 2021), and lacked adequate double blinding (Ross *et al.*, 2016) or an independent control group (Anderson *et al.*, 2020; DeKarske *et al.*, 2020; Ross *et al.*, 2021; Sage Therapeutics and Biogen, 2021a; Axsome Therapeutics, 2022). Additionally, the drugs of interest were often tested only as adjunctive therapy to the ongoing antidepressants (Clinicaltrials.gov, 2017; Brooks *et al.*, 2019; Fava *et al.*, 2019, 2022; VistaGen Therapeutics, 2019; Acadia, 2020; Savitz *et al.*, 2021; Sage Therapeutics and Biogen, 2022) and further trials should demonstrate whether the antidepressant effect is maintained in monotherapy. Most studies excluded patients having depression with mixed features, psychotic and substance use disorders, previous suicide attempts, or current suicidal ideation and/or significant medical comorbidities, thus limiting generalizability (Doss *et al.*, 2021; Tabuteau *et al.*, 2022). Several trials included only cases with moderate depression, thus limiting extrapolations to patients with severe depressive symptoms or TRD (Brooks *et al.*, 2019; Fava *et al.*, 2019; Gunduz-Bruce *et al.*, 2019; Monti *et al.*, 2019; Recourt *et al.*, 2019; Sage Therapeutics, 2019; VistaGen Therapeutics, 2019; Acadia, 2020; Carhart-Harris *et al.*, 2021; Doss *et al.*, 2021; Sage Therapeutics and Biogen, 2021b; 2021a; Savitz *et al.*, 2021; Sage Therapeutics and Biogen, 2022; Becker *et al.*, 2022; Iosifescu *et al.*, 2022; Praxis, 2022; Tabuteau *et al.*, 2022). Overall, patients were not fully representative of the real-world population, and this limitation should be considered for future studies.

Regarding psilocybin, it has been tested as part of structured psychotherapy. A therapeutic approach including psychotherapy requires a significant effort in terms of time and costs, with consequent possible limitations in the applicability to public health services (Doss *et al.*, 2021). Furthermore, it remains unclear whether psilocybin could have an antidepressant effect alone since there were no experimental conditions omitting supportive psychotherapy. The effects of micro-dosing should also be clarified, in relation to positive emotionality/beliefs and in reducing anxiety and depression compared to a single macro-dose.

Concerning some psilocybin trials, strategies to improve the recruitment of more diverse study populations are needed (Carhart-Harris *et al.*, 2021; Doss *et al.*, 2021; Ross *et al.*, 2021). The available evidence supports the efficacy of psilocybin in patients with cancer, but these results may not be generalizable to other end-of-life medical diseases (e.g. neurodegenerative diseases) or in severe pain conditions. Additionally, psilocybin, like other psychedelics, is known to promote suggestibility, which might have enhanced positive outcomes (Carhart-Harris *et al.*, 2015). Future studies should address the role of expectancy and suggestibility by measuring and controlling for these variables. Nonetheless, the approval of psilocybin remains extremely challenging due to the lack of appropriate ethical guidelines that can prevent its misuse and the consequent restrictions imposed by the Drug Enforcement Administration, despite no potential to cause addiction was found in the discussed trials. In this regard, there are no conflicts of interest for the authors of this manuscript.

Regarding seltorexant, although it showed benefits on residual insomnia in MDD, some trials were not primarily powered to demonstrate an antidepressant effect or efficacy on sleep disturbances (Brooks *et al.*, 2019; Recourt *et al.*, 2019).

The discussed drugs were all developed for nonparenteral administration. Considering the more robust and rapid effect of standard antidepressants when administered intravenously versus orally (Buoli *et al.*, 2019), future development of parental formulations (other than oral) should be considered. Although most trials assessed objective symptoms, the evaluation of subjective well being, functional endpoints, and quality of life would be important in future studies. Additionally, most clinical trials used the HDRS (Hamilton, 1960), Mongtomery-Asberg Rating Scale (Montgomery and Åsberg, 1979), or the Quick Inventory of Depressive Symptomatology–Self Report (Rush *et al.*, 2003); however, these instruments were designed to evaluate symptoms over a period of weeks; the use/development of different psychometric instruments to assess changes in symptoms severity within hours to days should be considered.

### Conclusion

Novel rapid acting antidepressants represent a promising option, particularly for patients unresponsive to standard medications or those who discontinue treatment due to side effects.

The lack of psychotomimetic, dissociative, metabolic, and rebound side effects greatly increases the safety and tolerability profile of new antidepressants, favouring a good therapeutic adherence; however, some side effects need longer follow-ups to be reliably assessed, such as weight gain and withdrawal symptoms.

The benefits of each compound on specific symptom domains/profiles were discussed, as this should be a key objective of clinical trials, to facilitate the implementation of precision psychiatry.

## Acknowledgements

This work was supported by #NEXTGENERATIONEU (NGEU) and funded by the Italian Ministry of University and Research, National Recovery and Resilience Plan, project MNESYS (PE0000006) – a multiscale integrated approach to the study of the nervous system in health and disease (DN. 1553 11.10.2022).

### Conflicts of interest

Prof A.S. is or has been a consultant to or has received honoraria or grants unrelated to the present work from Abbott, Abbvie, Angelini, Astra Zeneca, Clinical Data, Boheringer, Bristol Myers Squibb, Eli Lilly, GlaxoSmithKline, Innovapharma, Italfarmaco, Janssen, Lundbeck, Naurex, Pfizer, Polifarma, Sanofi, Servier, and Taliaz. Dr C.F. was a speaker for Janssen. For the remaining authors, there are no conflicts of interest.
